# A review of public comments submitted to the Centers for Medicare and Medicaid Services in response to the 2022 National Coverage Decision on treatment for Alzheimer’s disease

**DOI:** 10.1093/jlb/lsaf004

**Published:** 2025-04-04

**Authors:** Kaatje S Greenberg, Holly Fernandez Lynch, Chisom Nwakama, Jonathan Frumovitz, Samarth Setru, Ariel M Johnson, Saloni M Shah, Liliana Schadt, Matthew S McCoy, Allison K Hoffman, Emily A Largent

**Affiliations:** University of Pennsylvania Carey Law School, 3501 Sansom Street, Philadelphia, PA 19104, USA; Department of Medical Ethics and Health Policy, University of Pennsylvania Perelman School of Medicine, 423 Guardian Drive, Philadelphia, PA 19104, USA; University of Pennsylvania Carey Law School, 3501 Sansom Street, Philadelphia, PA 19104, USA; Department of Medical Ethics and Health Policy, University of Pennsylvania Perelman School of Medicine, 423 Guardian Drive, Philadelphia, PA 19104, USA; University of Pennsylvania School of Dental Medicine, 240 South 40th Street, Philadelphia, PA 19104, USA; University of Pennsylvania School of Dental Medicine, 240 South 40th Street, Philadelphia, PA 19104, USA; University of Pennsylvania School of Dental Medicine, 240 South 40th Street, Philadelphia, PA 19104, USA; University of Pennsylvania School of Nursing, 418 Curie Boulevard, Philadelphia, PA 19104, USA; Department of Medical Ethics and Health Policy, University of Pennsylvania Perelman School of Medicine, 423 Guardian Drive, Philadelphia, PA 19104, USA; University of Pennsylvania Carey Law School, 3501 Sansom Street, Philadelphia, PA 19104, USA; Department of Medical Ethics and Health Policy, University of Pennsylvania Perelman School of Medicine, 423 Guardian Drive, Philadelphia, PA 19104, USA

**Keywords:** Alzheimer's disease, drug approval, drug coverage, Medicare, public comments

## Abstract

Alzheimer’s disease (AD) is a neurodegenerative disease with devastating personal and social consequences. In June 2021, the U.S. Food and Drug Administration (FDA) granted accelerated approval to aducanumab (Aduhelm; Biogen), a first-in-class monoclonal antibody (mAb) for treatment of AD. In July 2021, responding to the significant controversy sparked by aducanumab’s approval, the Centers for Medicare and Medicaid Services (CMS) opened a National Coverage Determination (NCD) analysis for mAbs intended for the treatment of AD. CMS received a record number of public comments on the proposed NCD, which included a proposal for coverage with evidence development (CED). We undertook an in-depth qualitative analysis of those comments. Broad themes included: the appropriateness of FDA’s approval of aducanumab; the nature of the relationship between CMS and FDA; anticipated downstream effects of CED on innovation and health equity; aducanumab’s cost, value, and affordability; and whether aducanumab offered patients hope. The aducanumab controversy occurred at the intersection of multiple contentious issues; in the discussion, we contextualize our findings within these broader debates. Though Biogen pulled aducanumab from the market in early 2024, the effects of the public discourse surrounding its approval and coverage have been long-lasting and far-reaching, affecting health law, policy, and clinical practice.

## I. INTRODUCTION

Alzheimer’s is a neurodegenerative disease that causes disabling cognitive and functional impairments and, as a result, has devastating personal and social consequences.^1^ As the main cause of dementia, Alzheimer’s disease (AD) affects an estimated 6.7 million Americans and, due to associated care needs, millions more family caregivers.^2^ A dearth of proven treatment options has left many patients and caregivers eager for something that might slow the onset or progression of cognitive and functional decline.^3^

In June 2021, the U.S. Food and Drug Administration (FDA) announced it had granted accelerated approval to aducanumab (Aduhelm; Biogen), a first-in-class monoclonal antibody (mAb) that targets amyloid plaques in the brain.^4^ These plaques are a hallmark pathology of AD. Aducanumab was the first new Alzheimer’s treatment to enter the market in nearly two decades and the first ever intended to target the underlying disease process. Though aducanumab ostensibly represented an important advance for the field, its approval drew substantial criticism.^5^ Critics raised concerns about weak evidentiary support for the drug’s effectiveness, well-documented evidence of aducanumab’s serious risks, and its high cost.^6^ Overall, they worried about setting a dangerous regulatory precedent in AD and beyond.^7^

Medicare covers items and services that are reasonable and necessary for the diagnosis or treatment of illness or injury and that are within the scope of coverage that Medicare is statutorily authorized to provide.^8^ The ‘reasonable and necessary’ coverage standard is distinct from FDA’s ‘safe’ and ‘effective’ drug approval standard. Though divergence is unusual, FDA approval does not entitle a product to Medicare coverage.^9^ The Centers for Medicare and Medicaid Services (CMS) are authorized to make national coverage determinations (NCDs) that grant, limit, or exclude coverage to all Medicare beneficiaries.^10^ NCDs are made through a formal process, established by statute, that includes an assessment of clinical evidence as well as opportunities for public input. NCDs are binding on Medicare contractors, the private health insurers who process medical claims for Medicare beneficiaries.^11^ Absent an NCD, an item or service may be covered at the discretion of Medicare contractors based on a local coverage determination.^12^ Ninety percent of Medicare coverage determinations are established at the local level.^13^

In July 2021, just one month after the FDA granted accelerated approval to aducanumab, CMS opened an NCD analysis on mAbs directed against amyloid for treatment of AD,^14^ responding to the significant controversy sparked by aducanumab’s approval.^15^ After completing internal analysis, CMS published an initial proposal in January 2022 to cover such drugs, when FDA-approved, under an approach called Coverage with Evidence Development (CED).^16^ CED allows CMS to condition coverage of items and services ‘that are likely to show benefit for the Medicare population, but where the available evidence base does not provide a sufficiently persuasive basis for coverage outside the context of a clinical study.’^17^ When CED is employed, the agency will cover the item or service only in the context of an approved clinical study or when additional data are collected to assess appropriateness. CMS has noted that CED can be useful when interested parties’ expectations exceed the existing evidence.^18^

The proposed NCD was noteworthy for at least three reasons. First, its scope included aducanumab and all other drugs in its class, although aducanumab was the only FDA-approved drug of its kind at the time.^19^ Second, CMS rarely employs CED, which it began to formally implement in 2005.^20^ By the time CMS proposed CED for mAbs for the treatment of AD in early 2022, only 26 of 348 NCDs had adopted CED.^21^ On the rare occasions CED had been employed, it had generally been required for devices, diagnostics, or procedures rather than drugs or biologics. This is because evidentiary requirements for devices, diagnostics, and procedures to obtain market access are generally less stringent than for drugs and biologics; as a result, the former can be adopted into practice with relatively less evidence of benefit than the latter.^22^ Third, this was the first time CMS had proposed to cover a drug through CED with a randomized controlled trial (RCT) requirement.^23^

The proposal was open for public comments from January 11 to February 10, 2022.^24^ CMS received more than 10,000 comments, which it described as ‘a record number of comments on the proposed NCD and decision memorandum.’^25^ As an administrative agency, CMS is legally required to review and respond to public comments as part of its decision making. It need not accept the majority view or respond to each individual comment, but it must consider the thematic points raised by commentors and explain its decision in a manner that demonstrates the agency’s decision is neither arbitrary nor capricious.^26^

The public comment process recognizes that, although CMS decisions should be evidence-based, whether a drug is reasonable and necessary for the Medicare population is also partly a normative question. The putative value of public comments is that they can provide data, arguments, and insights relevant to CMS’s decision-making, including from patients and caregivers who have lived experience and will be directly affected by the agency’s decisions; patient advocacy groups who can offer broader insights beyond individual perspectives; clinicians who are tasked with helping patients navigate their treatment options and have experience interpreting and applying evidence about risks and benefits; industry representatives who have vested interests in the outcomes; and members of the public who may not have strong individual interests but could nevertheless be affected by wider implications of the decision, such as increased Medicare premiums.^27^ Thus, these comments complement the agency’s scientific and technical expertise, such that the public comment process can render decision making both more inclusive and more responsive.^28^ However, precisely how policymakers should balance scientific and technical data against potentially conflicting stakeholder perspectives remains unresolved, and there are no well-accepted measures for determining whether policy decisions are substantively improved through this sort of public engagement. Nonetheless, it is reasonable to expect both that stakeholder input can help policymakers identify blind spots in their analyses and that allowing those likely influenced by a policy decision to have the opportunity to share their perspectives is an appropriate procedural step.

With these considerations in mind, we undertook an in-depth analysis of the public comments submitted to CMS in response to its proposed NCD. These comments are publicly available on the CMS website. Unlike CMS, which used the comments specifically to inform its NCD, our aims were to characterize the claims and arguments advanced by different stakeholders and to understand how those tracked larger debates surrounding aducanumab’s approval and coverage. Importantly, the implications of this analysis extend beyond aducanumab itself.

Though ‘once billed as history’s greatest blockbuster drug, [aducanumab] ended up generating almost no income after insurers refused coverage and clinicians chose not to prescribe it.’^29^ Thus, it was not surprising when the drug maker Biogen announced in January 2024 that it would ‘discontinue the development and commercialization of ADUHELM’ and end the ENVISION clinical study, a post-marketing study designed to demonstrate clinical benefit and pave the way for traditional FDA approval.^30^ When announcing the discontinuation of aducanumab, Biogen also stated it would ‘continue to advance LEQEMBI … the first anti-amyloid beta treatment with FDA traditional approval in the United States.’^31^ Lecanemab (Leqembi; Biogen) was approved in January 2023 under the accelerated approval pathway and converted to traditional approval in July of that year.^32^ As of July 2024, the FDA has also granted traditional approval to donanemab (Kisunla; Lilly), another mAb that targets amyloid.^33^ Although both lecanemab and donanemab have demonstrated slowing of the progression of AD, the magnitude of the benefit is small and may not be discernable to patients and their families.^34^ Both drugs also have important safety risks.^35^

In one respect, Biogen’s decision to pull aducanumab from the market ‘close[d] out a yearslong saga that generated outrage and eroded trust in the regulatory process for bringing new drugs to market.’^36^ In another respect, aducanumab continues to play an outsized role. The NCD it precipitated remains in effect for all drugs in the class, and the reputational effects for FDA and CMS linger. Further, aducanumab informed ongoing policy debates, including whether payer coverage of FDA-approved drugs should be assumed (as it traditionally has been), whether coverage should be adjusted downward for drugs granted accelerated approval, and what the role of payers should be in promoting evidence generation beyond FDA approval.^37^ Thus, while aducanumab is no longer on the market, the public comments on the proposed NCD remain worthy of study.

In Section II, we provide additional background on aducanumab and the proposed NCD. In Section III, we describe our qualitative methods. Our results are presented in Section IV. We look at who submitted (and did not submit) comments to CMS and compare and contrast the content of unique comments—those that appeared only once in the record—and duplicate comments—‘form letters, where at least part of the comment contained nearly identical language.’^38^ As in broader public discussions surrounding aducanumab, the comments reflected that CMS’s decision to drastically restrict coverage of this drug class was both lauded as a necessary check on FDA’s controversial approval^39^ and criticized as restricting access to potentially life changing treatments and infringing on FDA authority.^40^ In Section V, we discuss the results, situating them within broader health law and policy debates while also providing updates on subsequent developments.

## II. BACKGROUND

The story of aducanumab’s rocky path to FDA approval has been told elsewhere.^41^ Here, we offer a brief overview to help contextualize the comments submitted to CMS.

On March 21, 2019, Biogen cited futility as its reason for the early stopping of a pair of Phase III clinical trials evaluating aducanumab’s effectiveness on a clinical endpoint: slowing cognitive impairment as measured by changes in the Clinical Dementia Rating-Sum of Boxes (CDR-SB) score.^42^ Thus, many found it surprising when, in October 2019, Biogen announced it had reviewed a larger dataset, conducted additional analyses,^43^ and planned to pursue FDA approval.^44^

At a November 2020 meeting of the FDA’s Peripheral and Central Nervous System Advisory Committee, the committee’s external experts resoundingly voted that the evidence presented did not support aducanumab’s effectiveness.^45^ Members pointed to the fact that aducanumab failed to meet clinical endpoints in pre-specified analyses, particularly slowing cognitive impairment, while posing substantial risk of brain bleeds and swelling.^46^

The Advisory Committee did not discuss whether aducanumab should be considered for accelerated approval because a senior FDA official indicated the agency was not considering that approach.^47^ Nor did the committee discus the suitability of beta-amyloid plaque in the brain as a surrogate endpoint.^48^ Unlike traditional approval based on evidence of effectiveness supported by clinical endpoints that directly assess how a patient feels, functions, or survives, the accelerated approval pathway allows the FDA to approve drugs for serious conditions that fill an unmet medical need based on unvalidated surrogate endpoints deemed reasonably likely to predict clinical benefit, but not yet confirmed to do so.^49^ The FDA requires sponsors to conduct post-approval studies to confirm the benefit of accelerated approval drugs, but those studies face a number of problems, leading some to question whether access and evidence are being appropriately balanced.^50^ Still, patients often find the accelerated approval pathway attractive, as it may provide earlier access to a drug than would otherwise be possible.^51^ Early access is also lucrative for drug sponsors; drugs granted accelerated approval by the FDA account for substantial CMS spending.^52^

In light of comments made to the Advisory Committee by FDA officials, it came as a shock when the FDA subsequently announced aducanumab’s accelerated approval based on the drug’s successful reduction of beta-amyloid plaque in the brain, an unvalidated surrogate endpoint never before used to support drug approval.^53^ Use of amyloid reduction as a surrogate endpoint was particularly controversial because, even before Biogen halted its Phase III aducanumab trials, multiple other Phase III trials of anti-amyloid therapies had failed to show clinical benefit. Further, Alzheimer’s researchers have noted that the number of amyloid deposits in the brain is not well correlated with patients’ degree of cognitive impairment.^54^ Several Advisory Committee members published an article in the New  England  Journal of  Medicine that provides both helpful background information and a review of the approval decision, concluding that it reflected a ‘regulatory failure’ and predicting that ‘the decision will reverberate for years.’^55^

Publicly justifying its decision to grant accelerated approval to aducanumab, the FDA pointed to AD patients’ and caregivers’ perceived willingness ‘to accept the trade-off of some uncertainty about clinical benefit in exchange for earlier access to a potentially effective drug … .’^56^ Critics, meanwhile, cited the scant evidence of clinical benefit, substantial safety concerns, and fear of establishing problematic precedent. Additionally, they warned that aducanumab’s controversial approval risked erosion of trust in the FDA’s approval mechanisms^57^ and raised concerns about regulatory capture.^58^

Once aducanumab was approved, attention turned to the drug’s price, which was initially $56,000 per year (exclusive of the substantial costs of patient monitoring). This price tag far exceeded the $3000 to $8400 per year that the Institute for Clinical and Economic Review (ICER)—an independent research organization that examines the clinical and economic value of prescription drugs, devices, and medical tests—concluded would constitute a fair price.^59^ Because most people diagnosed with AD are over the age of 65 and thus are eligible for Medicare, much attention was focused on CMS and whether it would pay for aducanumab. Biogen, which anticipated a potential patient population of 1–2 million for its drug, estimated that 80% of patients would be covered through Medicare Part B (medical insurance); ICER explained that would make aducanumab ‘the cause for potentially doubling the $37 billion Medicare currently spends each year on all Part B drugs combined.’^60^ In November 2021, CMS announced it was raising Part B premiums for all enrollees to cover the anticipated costs of aducanumab.^61^ The next month, reacting to substantial criticism and low uptake of the drug, Biogen announced it was reducing aducanumab’s cost per year from $56,000 to $28,000. At the time, Biogen’s CEO explained that ‘[Biogen had] listened to the feedback of [its] stakeholders, and [it was] now taking important actions to improve patient access to ADUHELM.’^62^

Both the increase in Part B premiums and Biogen’s price reduction were announced after CMS opened its NCD analysis on mAbs directed against amyloid for treatment of AD in July 2021.^63^ At that time, coverage determinations were ‘being made at the local level by Medicare Administrative Contractors who represent 12 jurisdictions across the country.’^64^ CMS opened the NCD to determine whether a national policy was appropriate.^65^ Under the NCD process, CMS seeks ‘to determine whether or not an unbiased interpretation of the available evidence base supports or refutes the requested coverage in whole or in part.’^66^ NCDs occur through an evidence-based process with opportunities for public participation, including a 30-day public comment period.^67^ The agency may obtain additional input through technology assessments from outside entities and/or deliberation by the Medicare Evidence Development and Coverage Advisory Committee.^68^

In January 2022, although aducanumab was then the only FDA-approved drug of its kind, CMS published an initial proposal to cover all FDA-approved mAbs directed against amyloid for treatment of AD under CED.^69^ The agency’s authority to employ CED is based on its interpretation of Sections 1862(a)(1)(A) and 1862(a)(1)(E) of the Social Security Act, which allow payment for items and services deemed *not* reasonable and necessary if they are being used for research or in other limited circumstances.^70^ Section 1142 of the Social Security Act further authorizes CMS to conduct and support research that appropriately reflects the needs and priorities of the Medicare program.^71^

CMS’s January 2022 CED proposal would have conditioned coverage for Medicare beneficiaries on several criteria. Consistent with aducanumab’s FDA-approved label, CMS proposed to cover mAbs directed against amyloid for treatment of AD only for patients with either a clinical diagnosis of mild cognitive impairment (MCI) due to AD or mild AD dementia and also evidence of amyloid pathology consistent with AD.^72^ Patients with any other neurological or medical condition that could significantly contribute to cognitive decline were excluded from the coverage criteria.^73^ This effectively barred coverage for individuals with Down Syndrome, 50% of whom develop AD by their 60s,^74^ an exclusion criterion that was especially noteworthy because many adults with Down Syndrome, regardless of their age, are eligible for Medicare.^75^ (Medicaid is also a major insurance provider for this population.^76^) CMS also proposed limiting approved clinical trials to hospital-based outpatient facilities.^77^

Members of the public submitted over 10,000 comments to CMS during the comment period, which ran from January 11 to February 10, 2022; CMS published 9957 comments that did not include excessive personal health information to its website.^78^ The agency noted that more than half of the comments were duplicates.^79^ Duplicates are collections of identical or nearly identical comments sponsored by various organizations and submitted by the group’s members or supporters.^80^ In its final decision memo, CMS identified 10 broad themes it drew from the public comments, some of which were further broken down into subtopics, as summarized in Table 1**.**

**Table 1:** CMS’s Summary of key themes covered in PUBLIC COMMENTS on the proposed National Coverage Determination (NCD)^*^

Lack of Evidence for Clinical Benefit	• The majority of public comments stated that there is not enough evidence, as of Spring 2022, showing that these drugs/biologicals provide a clinical benefit in order to cover antiamyloid monoclonal antibodies (mAbs).
Antiamyloid mAbs Approved for Treatment of Alzheimer’s Disease (AD) Based on a Surrogate Endpoint Considered Reasonably Likely to Predict Clinical Benefit	• Several commenters stated that CMS should not restrict access to a U.S. Food and Drug Administration (FDA) approved treatment for AD, especially since there are no alternative options available.
Coverage with Evidence Development (CED) Randomized Controlled Trial (RCT) Requirement	• Several commenters stated that the RCT requirement for a drug that has been FDA-approved is unprecedented, with some questioning CMS’ authority for such a requirement. • Many commenters suggested covering antiamyloid mAbs only in RCTs is duplicative of trials required by FDA designed to answer the question of clinical benefit. • Many commenters did not agree with the requirement that all trials must be conducted in a hospital-based outpatient center, stating that this would further restrict access for patients in underserved areas and discriminate against independent outpatient infusion centers. • Some commenters stated that the CED requirement would hurt future innovation for AD treatments.
Antiamyloid mAbs as a Class	• Many commenters stated that the proposed CED NCD should be specific to Aduhelm only. • Some commenters suggested that a decision for the entire class of drugs/biologics is premature given the antiamyloid mAbs that are currently in development. • Some commenters recommended that the CMS coverage decision should be limited to only antiamyloid mAbs that are approved under the FDA accelerated approval pathway. An additional randomized controlled trial, outside of the FDA required confirmatory trial, should not be a Medicare requirement. • Public commenters stated that if an antiamyloid mAb demonstrates a meaningful clinical benefit, future antiamyloid mAbs should be covered. • Some commenters suggested alternative study designs that are less rigorous than RCTs, such as observational studies.
Health Equity	• Many commenters were supportive of the requirement in the proposed decision which specified that the diversity of patients included in each trial must be representative of the national population diagnosed with AD. • Commenters mentioned that while the proposed decision specifies a diversity requirement, only covering antiamyloid mAbs in RCTs would actually worsen health equity issues due to the relatively small number of patients that would be enrolled in these trials, and the location of study sites away from rural areas. • Commenters stated that a CED decision would discriminate against patients with AD because CMS has previously covered treatments for conditions such as HIV and cancer that had been approved by the FDA under accelerated approval.
Down Syndrome	• There were a large number of public comments, ~1800, that requested beneficiaries with Down syndrome and other intellectual and developmental disabilities (IDD) have the same coverage other patients would have to antiamyloid mAbs.
PET for Beta Amyloid	• Some commenters expressed concern over the specification in the proposed decision that one beta amyloid PET scan will be covered, if the patient did not previously receive a beta amyloid PET scan. These commenters were aware that this restriction is due to the CED requirements included in the Beta Amyloid Positron Emission Tomography in Dementia and Neurodegenerative Disease NCD (220.6.20), but ask CMS to reconsider this limitation with the final NCD for antiamyloid mAbs.
Part B Premiums	• Many commenters expressed their concern with the price of Aduhelm and frustration with the increase in Medicare premiums, stating that Medicare should negotiate the price for these drugs.
Medicaid	• A couple of commenters expressed concern over the proposed decision stating that the restrictiveness of CED would shift coverage of antiamyloid mAbs for the treatment of AD from Medicare to Medicaid.

^*^We adapted this from https://www.cms.gov/medicare-coverage-database/view/ncacal-decision-memo.aspx?proposed=N&ncaid=305 and lightly edited for length and clarity.

Ultimately, CMS retained the CED approach in its final rule, though with important modifications. It limited Medicare coverage of FDA-approved mAbs directed against amyloid for treatment of AD to three circumstances: (i) for use according to the FDA-approved indication in National Institutes of Health (NIH)-supported trials; (ii) for drugs granted accelerated approval, use in RCTs conducted under an investigational new drug (IND) application; or (iii) for drugs granted traditional approval, CMS-approved ‘prospective comparative studies,’ which may include a registry or registry-based studies.^81^ Outside of these conditions, CMS will not cover these drugs. CMS noted that there ‘may be subpopulations that are important to study such as individuals with Down syndrome’ and that it supported ‘NIH and other federal agency trials in a broad range of clinical topics, which may include patients with Down syndrome.’^82^ It also removed the restriction that all trials had to be conducted in a hospital-based outpatient center.^83^

## III. METHODS

Because these comments were made publicly available on the CMS website and were not obtained through interaction or intervention with commentors, our analysis did not constitute human subjects research and was not subject to institutional review board oversight.

Scholars should assess how their own experiences and positions might contribute to their interpretations of others’ claims and experiences. With this in mind, we note that our research team was composed of members with diverse disciplinary backgrounds, including medical ethics, health policy, health law, political science, and patient care (nursing and dentistry); it included both faculty and student researchers.

### III.A. Data Collection

The data presented here were drawn from the 9957 comments submitted during the public comment period that were made available on CMS’s website.^84^ Each comment was accompanied by the commentor’s name and, when provided, a title or organizational affiliation. Comments ranged in length from a single sentence to multiple pages. The comments were downloaded from the CMS website in the summer of 2022, after the comment period closed.

### III.B. Qualitative Content Analysis

A qualitative content analysis approach was chosen for this project because our central goal was to characterize public opinion regarding CMS’s coverage determination—particularly to understand who was commenting and what arguments they made—not to generate an explanatory theory. Four authors (HFL, AH, MM, EAL) independently annotated 50 comments to identify themes; they then met to discuss these themes as well as key points from the literature relevant to aducanumab’s approval and the proposed NCD. These were formalized in a preliminary codebook, or taxonomy for categorizing qualitative data.^85^

CMS identified duplicates but did not provide its methods for doing so, requiring us to develop our own methodology. Our approach was inclusive: we did not require verbatim text but rather focused on key words and phrases. We considered comments to be duplicates if there were more than five comments sharing substantially similar or identical wording and phrases. This choice reflects our impression that, when key words or phrases were used by two to five individuals, they did not reflect an effort by a sponsoring organization to mobilize its members and supporters but instead reflected the comments developed by a family or similar small group. Three authors (SMS, SPS, AJ), working in parallel, reviewed the same subset of 100 comments to ensure a unified approach to identifying and recording duplicate comments. SMS, SPS, and AJ then each reviewed one-third of the remaining comments in the dataset (divided alphabetically) to identify and record duplicates. Each duplicate type was assigned a tag that was, along with the defining wording or phrases, included in the codebook.

Three authors (SMS, SPS, AJ) used the codebook to generate a Google form (available upon request) to facilitate review and analysis of the large quantity of data. This form contained a mix of questions about the commentor’s demographics, including their name, organization, and stakeholder type (eg physician or caregiver), as well a checklist to apply any relevant code(s). If a code was noted as present, the relevant text from the comment was copied and pasted into a text box for subsequent analysis.

Under the supervision of HFL and EL, three authors (SMS, SPS, AJ) double-coded a subset of 270 comments and assessed inter-coder reliability. They met regularly to compare their entries, discuss discrepancies, and refine the codebook to reduce ambiguity, eliminate redundancy, and enhance comprehensiveness. Changes were made to the Google form as needed to reflect changes in the codebook. Codebook revisions were applied to previously coded comments. As additional individuals (KG, LS, CN, JF) joined the research team, they were paired with trained coders and went through the process of double-coding entries to ensure familiarity with the Google form and consistent application of the codes. Having developed a refined codebook and agreement on its use, several authors (KG, LS, CN, and JF) single-coded the remaining comments; duplicates were marked as such to allow them to be analyzed separately from the unique comments.

After initial coding was complete, the results were exported into a Google sheet for analysis. We explored themes and findings, as well as interrelationships between themes and the association of themes with stakeholder types. This process was first undertaken by pairs of authors focused on select codes, then expanded to the full author group to refine ideas and arrive at the best description of our data. Because qualitative data are the result of an interpretive process,^86^ our general approach was *not* to quantify thematic patterns we found too nuanced to be amenable to the kind of categorization quantification would require. Rather than using specific numbers to suggest greater accuracy of the results than is justified by our methods,^87^ and as is common in qualitative research, we typically opted to characterize frequency using generalized approximations.

## IV. RESULTS

Of the 9957 published comments, only one-third (3009) included information from which we could infer the commentor’s stakeholder group affiliation(s). Overall, individual commentors commonly self-identified as (from most to least frequent): family members and caregivers of people with Down Syndrome; clinicians and other health care workers; family members and caregivers of people living with Alzheimer’s; Medicare beneficiaries; and academics and researchers (Table 2). Institutions and organizations, though less numerous, also submitted comments. The large number of family members and caregivers of people with Down Syndrome reflects a coordinated effort by Down Syndrome patient advocacy organizations to mobilize supporters, as discussed in the section on duplicate comments (§ IV.B) below.

**Table 2:** Stakeholder groups represented among commentors^*^

	All Comments N (%) N = 9957	Unique Comments N (%) N = 3501	Duplicate Comments N (%) N = 6456
Person living with Alzheimer’s disease (AD)	7 (0.1%)	6 (0.2%)	1 (0.02%)
Family member or caregiver of person with AD	500 (5.0%)	374 (10.7%)	126 (2.0%)
Person living with Down Syndrome (DS)	13 (0.1%)	5 (0.1%)	8 (0.1%)
Family member or caregiver of person with DS	1022 (10.3%)	413 (11.8%)	609 (9.4%)
AD patient advocacy organization	31 (0.3%)	27 (0.8%)	4 (0.1%)
Other advocacy organization	179 (1.8%)	118 (3.4%)	61 (0.9%)
Clinician or other health care worker	643 (6.5%)	450 (12.9%)	193 (3.0%)
Clinical society/organization	25 (0.3%)	22 (0.6%)	3 (0.1%)
Pharmaceutical company/industry association	29 (0.3%)	27 (0.8%)	2 (0.03%)
Insurer or other payer	6 (0.1%)	5 (0.1%)	1 (0.02%)
Medicare beneficiary	333 (3.3%)	191 (5.5%)	142 (2.2%)
Self-identified member of the general public/taxpayer	140 (1.4%)	84 (2.4%)	56 (0.9%)
Academic (including researchers)	231 (2.3%)	165 (4.7%)	66 (1.0%)
Other	193 (1.9%)	107 (3.1%)	86 (1.3%)
Not specified	6948 (69.8%)	1748 (49.9%)	5200 (80.5%)

^*^Commentors could be identified as belonging to more than 1 stakeholder group.

Across all commentors, there was broad agreement that AD has a terrible impact on patients and families. This was exemplified by comments calling Alzheimer’s a ‘cruel and awful’ disease or noting that AD threatens people with both ‘the very real and very frightening prospect of losing their minds’ and also a ‘slow horrible death.’ Commentors spoke to the pain of watching individuals inhabit ‘the world of Alzheimer’s [sic], healthy of body but lost in the fog of a disease that steals memories and the warmth of companionship.’ Commentors also noted the far-reaching consequences of a disease that can, as one commentor explained, wreak ‘havoc on [the] finances, … family dynamics and relationships, … careers, … and … mental and spiritual health’ of both patients and their caregivers. Further, many commentors acknowledged that AD is characterized by inequity—in who bears the burden of disease, in who has access to memory care, and in who participates in research. Indeed, the awfulness of the disease and the pervasiveness of disparities may be seen as stipulated facts, agreed to by all parties.

As shown in Table 3, just over two-thirds of all comments addressed CMS’s planned use of CED, a key feature of the proposed NCD. Amongst those commentors who mentioned it, roughly 10% favored CED, while less than 10% rejected CED in favor of full coverage. The remaining commentors mentioning CED, just over 80%, objected to *any* Medicare coverage of mAbs directed against amyloid for treatment of AD, with or without CED. This strong overall opposition to any coverage should not obscure notable variation within and between stakeholder groups. For instance, most commentors who reported an affiliation with an AD patient advocacy organization and nearly half of individuals who self-identified as a family member or caregiver for a person with AD favored full coverage. Clinicians and other health care workers, as well as academics and researchers, were almost evenly divided between favoring CED and opposing any coverage at all, with very few supporting full coverage. Meanwhile, the majority of commentors who objected to any coverage submitted text from a form letter (discussed in § IV.B below) and did not self-identify as belonging to any particular stakeholder group(s).

**Table 3:** Views on CED by stakeholder type, duplicate vs unique comments

**Stakeholder type** ^*^	**Duplicate comments**				**Unique comments**			
	**Number of stakeholders addressing CED** **N = 5279** ^**^	**Of those stakeholders who expressed any opinion…**			**Number of stakeholders addressing CED** **N = 1529**	**Of those stakeholders who expressed any opinion…**		
		**Oppose any coverage** **N (%)** **N = 5108**	**Favor CED** **N (%)** **N = 144**	**Favor full coverage** **N (%)** **N = 27**		**Oppose any coverage** **N (%)** **N = 479**	**Favor CED** **N (%)** **N = 571**	**Favor full coverage** **N (%)** **N = 479**
Person living with Alzheimer’s disease (AD)	1	1 (100.0%)	0 (0.0%)	0 (0%)	4	0 (0.0%)	1 (25.0%)	3 (75.0%)
Family member or caregiver of person with AD	79	65 (82.3%)	7 (8.9%)	7 (8.9%)	226	36 (15.9%)	55 (24.3%)	135 (59.7%)
Person living with Down syndrome (DS)	0	–	–	–	0	–	–	–
Family member or caregiver of person with DS	0	–	–	–	3	0 (0.0%)	1 (33.3%)	2 (66.7%)
AD patient advocacy organization	3	0 (0.0%)	1 (33.3%)	2 (66.7%)	21	1 (4.8%)	2 (9.5%)	18 (85.7%)
Other advocacy organization	15	11 (73.3%)	4 (26.7%)	0 (0.0%)	48	5 (10.4%)	22 (45.8%)	21 (43.8%)
Clinicians and other health care workers	149	93 (62.4%)	53 (35.6%)	3 (2.0%)	277	68 (24.5%)	136 (49.1%)	73 (26.4%)
Clinical society or organization	3	3 (100.0%)	0 (0.0%)	0 (0.0%)	17	2 (11.8%)	9 (52.9%)	6 (35.3%)
Pharmaceutical company or industry association	1	1 (100.0%)	0 (0.0%)	0 (0.0%)	14	0 (0.0%)	5 (35.7%)	9 (64.3%)
Insurer or other payer	1	1 (100.0%)	0 (0.0%)	0 (0.0%)	4	0 (0.0%)	4 (100.0%)	0 (0.0%)
Medicare beneficiary	136	120 (88.2%)	16 (11.8%)	0 (0.0%)	96	53 (55.2%)	35 (36.5%)	8 (8.3%)
Self-identified member of the general public or taxpayer	50	48 (96.0%)	1 (2.0%)	1 (2.0%)	48	19 (39.6%)	21 (43.8%)	8 (16.7%)
Academic (including researchers)	64	58 (90.6%)	6 (9.4%)	0 (0.0%)	102	21 (20.6%)	66 (64.7%)	15 (14.7%)
Other	55	50 (90.9%)	3 (5.5%)	2 (3.6%)	59	11 (18.6%)	22 (37.3%)	26 (44.1%)
Not specified/not sure	4722	4657 (98.6%)	53 (1.1%)	12 (0.3%)	610	263 (43.1%)	192 (31.5%)	155 (25.4%)

^*^Commentors could be identified as belonging to more than 1 stakeholder group.

^**^Many commenters expressed no opinion on CED.

In what follows, we report on the comments in greater depth, addressing the content of the 3501 unique comments (§ IV.A) before turning to the duplicate comments (§ IV.B).

### IV.A. Unique Comments

The unique comments reflected their authors’ diverse viewpoints. While some commentors brought relevant lived experience to bear (eg family members or caregivers for persons with AD), others had relevant professional expertise (eg clinicians and academics), or a guiding organizational viewpoint (eg patient advocacy organizations or pharmaceutical companies). Many commentors disclaimed any specific interest in AD but nevertheless felt invested in the outcome as members of the public (eg as taxpayers or Medicare beneficiaries). Comments reflected varying levels of knowledge and sophistication about topics ranging from Alzheimer’s to FDA approval processes to health care financing.

#### 1. Unique Commentors’ Views on CED

Of the 3501 total unique comments, roughly one-third took a position on CED (Table 3), despite that being a focus of the public comment period. Within this subset addressing CED, roughly equal numbers favored CMS’s proposal to use CED, favored full coverage of aducanumab (ie, no CED), or opposed any coverage at all of mAbs directed against amyloid for treatment of AD. A few urged CMS to limit the NCD to aducanumab, rather than extending it to all FDA-approved drugs in the class.

More than half of the commentors who favored CED expressed uncertainty about the safety or efficacy of aducanumab or the soundness of aducanumab’s FDA approval process. Many explicitly commended CMS for utilizing CED to limit coverage. In order of frequency, the most common rationales included in comments favoring CED were: uncertainty about aducanumab’s benefits; concern about aducanumab’s affordability; the perceived importance of confirming aducanumab’s safety and efficacy; and, to a lesser extent, a belief that FDA should not have approved aducanumab at all.

Among commentors who rejected CED and sought full coverage, nearly two-thirds expressed a desire to broaden patients’ access to aducanumab. These commentors often discussed their own experiences or those of family members or acquaintances with AD who have, as one commenter put it, ‘no more time for debate or delay.’ Beyond access, many commentors mentioned one or more of the following rationales for full coverage, in order of frequency: the importance of sustaining patient optimism and hope; a lack of alternative treatment options; and a perceived ‘right to try’ a promising drug. Several also expressed concerns that CMS would be overstepping its statutory authority by limiting coverage of an FDA-approved drug and that CMS’s decision to limit coverage could have an unfavorable impact on drug development going forward.

Of those commentors who opposed any coverage, with or without CED, more than half focused on concerns about aducanumab’s cost, value, and/or affordability, while just under half raised worries about aducanumab’s uncertain clinical benefits. Roughly one-third said that the FDA should not have approved aducanumab, with a handful also expressing worries specifically about the accelerated approval pathway or criticizing FDA or CMS for creating false hope by approving or covering a potentially ineffective drug.

The remaining two-thirds of unique comments—those that did not explicitly address CED—expounded on topics similar to those just outlined. Many commentors expressed concern or even anger at aducanumab’s high price. Some urged CMS to address the drug’s costs, whereas others voiced frustration at the perceived greed of Biogen or of the pharmaceutical industry more broadly. Still more simply shared stories of family members diagnosed with AD.

In the sections that follow, we explore seven cross-cutting themes in greater detail. In §IV.A.2, we examine unique commentors’ overall views on the FDA’s approval of aducanumab as well as sub-themes on the adequacy of the evidence of safety and efficacy and use of the accelerated approval pathway. In §IV.A.3, we show how the handling of aducanumab shaped commentors’ perspectives of CMS and FDA, as well as the relationship between them. In §IV.A.4, we explore commentors’ thoughts on the downstream effects of CED on drug development and evidence generation. In §IV.A.5, we describe comments that addressed the potential downstream effects of the proposed NCD on health equity. In §IV.A.6, we characterize comments on aducanumab’s cost, value, and affordability. This section includes sub-themes on how aducanumab will affect Medicare and Medicaid, the opportunity costs incurred by spending money on aducanumab, pharmaceutical industry greed, and the need for CMS to be able to negotiate drug prices. In §IV.A.7, we consider comments that depict aducanumab as either a source of hope or a source of false hope. Finally, in §IV.A.8, we examine comments on importance of giving patients a choice about whether or not to take it.

#### 2. FDA’s Approval of Aducanumab

About one in 10 of the unique comments addressed FDA’s decision to approve aducanumab (Table 4). Some deemed FDA’s decision reasonable. For instance, one commentor explained that ‘[a]nything that is FDA approved has already been carefully considered’ while another lauded the agency’s ‘expert, scientifically-driven decision-making process.’

**Table 4:** Exemplary quotes regarding FDA approval and use of the accelerated approval pathway

**When FDA approved aducanumab, it …**	
*… made the right decision.*	• ‘Ultimately, the sole authority to approve Aduhelm based on the degree to which it reduced beta amyloid plaque in AD patients was the FDA’s. GAP firmly believes that this decision was well grounded, reasonable, and overwhelming good for patients.’ *(Alzheimer’s patient advocacy organization)* • ‘Furthermore, analysis of two of the trials DID SHOW modest clinical benefit, hence the FDA’s ultimate approval of the drug.’ *(Clinician or other health care worker)*
*… made the wrong decision.*	• ‘As a retired physician as well as caretaker of a [PHI Redacted] who lived with dementia for 12 years, I was appalled at the FDA decision to approve this drug in the face of a lack of evidence for its clinical effectiveness. There is essentially no good evidence that this drug helps.’ *(Family member/caregiver of person living with Alzheimer’s disease; Clinician or other health care worker)* • ‘This drug does not show clinical benefits. The CMS decision will limit the potential harms. Aduhelm (aducanumab) should have never been approved in the first place, but this decision from CMS will protect the public from access to an ineffective and dangerous drug.’ *(Clinician or other health care worker)*
**FDA’s use of the accelerated approval pathway was …**	
*… appropriate/justified.*	• ‘I feel that the FDA made the right decision at the time the decision was made. The follow-up clinical trial and history well tell us if this was the right thing for AD patients, just as similar opportunities have been given to cancer patients in previous FDA accelerated approval decisions.’ *(Clinician or other health care worker)* • ‘The Food and Drug Administration’s (FDA) use of the accelerated approval pathway (AAP) to approve Aduhelm for the treatment of AD was an informed and appropriate exercise of the authority vested in the FDA by Congress.’ *(Alzheimer’s patient advocacy organization)*
*… inappropriate/unjustified.*	• ‘There is no justification to approve or to pretend the trial did not happen and approved on the accelerated approval pathway. That is not the back-up if a trial fails. … There is no expert consensus that that plaque reduction coincides with some slowing of cognitive decline. In fact, the evidence to date is to the contrary.’ *(Family member/caregiver of person living with Alzheimer’s disease)* • ‘The FDA’s accelerated approval of this drug also appears not to have taken into account the significant health risks and side effects that are likely to arise for patients on this drug in exchange for a minimal or nonexistent benefit.’ *(Self-identified member of the general public/taxpayer)*

Most commentors, though, felt ‘the FDA should not have approved’ aducanumab. Multiple comments quoted Aaron Kesselheim, who had resigned from his position on the Peripheral and Central Nervous System Advisory Committee after aducanumab was approved and who publicly stated that this was ‘probably the worst drug approval decision in recent U.S. history.’^88^ Many commentors did not simply disagree with the approval of aducanumab, they also questioned the process by which FDA reached its decision, declaring it ‘flawed’ or ‘suspect at best.’ Clinicians and other healthcare workers were especially likely to express such concerns. The agency’s failure to ‘maintain[] [its] high standards,’ some commentors suggested, was untoward and a disservice not only to AD patients but to all who rely on the agency to ‘only approve treatments that are safe and effective.’ Other commentors characterized FDA’s approval of aducanumab as ‘a self-inflicted wound’ that could ‘set an abysmally low standard for new drug approvals … [and] consign[] the FDA to irrelevance.’ In addition to diminishing its own authority, commentors suggested that FDA’s approval of aducanumab ‘clearly diminished the Public’s [sic] trust in the FDA to put patients and the public first.’

About one-third of the commentors who disagreed with the FDA’s decision to approve aducanumab supported CED. As an example, one commentor wrote, ‘Aduhelm (aducanumab) should have never been approved in the first place, but this decision from CMS will protect the public from access to an ineffective and dangerous drug.’ Another third of commentors who disagreed with FDA’s approval decision were opposed to any Medicare coverage at all. We discuss the interplay of CMS and FDA decisions further in §IV.A.3.

##### i. Adequacy of the Evidence that Aducanumab is Safe and Effective

A substantial number of unique comments addressed the adequacy of evidence that aducanumab is safe and effective (Table 5). Within these comments, the majority perspective was that aducanumab’s overall risk–benefit profile was likely unfavorable. These commentors felt it was more likely than not that aducanumab simply ‘does not work’ or, somewhat more favorably, that the benefits of taking aducanumab were ‘uncertain.’ Reflecting on the evidence of safety, many of these commenters asserted that aducanumab is associated with significant risks, particularly risks of brain bleeding and swelling. For example, a comment jointly submitted by a collection of advocacy organizations cited a medical journal to support the claim that aducanumab ‘did cause brain swelling, brain bleeds, nausea, headaches, dizziness, confusion, and an altered mental state in approximately half of patients.’

**Table 5:** Exemplary quotes regarding adequacy of the evidence that aducanumab is safe and effective

**Based on the current evidence, it is reasonable …**	
*… to conclude aducanumab is safe and effective.*	• ‘There is reasonable evidence suggesting a clinical benefit in slowing symptomatic and functional decline which is supported by biomarker findings and initial results of similar drugs in its class that are currently in clinical trials.’ (*Clinician or other health care worker)* • `Biogen disagrees with the statement in the proposed NCD that no trial has been able to demonstrate a clinically meaningful improvement in patient health outcomes. FDA concluded that the EMERGE study “demonstrated a clinically meaningful and statistically significant treatment effect for the high dose of aducanumab on an accepted primary endpoint, the [Clinical Dementia Rating Sum of the Boxes scale (CDR-SB)], and across multiple secondary and tertiary endpoints.” ... CMS’ proposal does not offer a reasoned explanation for disagreement with FDA’s determination regarding the clinical benefit of ADUHELM.' *(Pharmaceutical company/Industry association)*
*… to conclude aducanumab is not safe or effective.*	• ‘[T]he data for aducanumab in phase three trials is extremely poor. It would appear that, more likely than not, the medicine provides no clinical benefits and has high potential for serious side effects of brain swelling and brain bleeding.’ (*Clinician or other health care worker)* • ‘Solloway and colleagues discuss harmful effects of Aduhelm in the JAMA Neurology Journal where results from Aduhelm trials show 41% of patients experienced brain swelling or bleeding when given the FDA approved dose of the drug. Given the overwhelming evidence that Aduhelm is minimally effective at best, why is there such a large push to approve this drug?’ *(Academic (including researchers))*
*… to gather more evidence.*	• ‘We strongly agree with CMS that there is insufficient evidence regarding the risks and benefits of aducanumab, and of amyloid targeting therapies in general, and agree that CMS should not provide coverage until there is clear evidence of safety and efficacy with clinically meaningful outcomes in a diverse patient population.’ *(Clinician or other health care worker; Academic (including researchers))* • ‘CMS has raised important questions about the available evidence on mAbs for AD, and in particular about the effectiveness of aducanumab, … to support a reasonable and necessary coverage determination for Medicare beneficiaries with MCI and early-stage AD. We agree that there are important evidentiary questions.’ *(Academic (including researchers))*
**Even if more research is conducted, …**	
*… CMS shouldn’t be funding it.*	• ‘How can Medicare justify paying for an incredibly expensive drug to be used in CLINICAL TRIALS? ... [T]he government and Medicare users should not be subsidizing clinical trials for Biogen by paying for this. Biogen should pay ... rather than giving Biogen a huge profit on every dose—for them to CONTINUE a clinical trial which should have already been completed before submission to the FDA.’ *(Not specified)* • ‘Why can’t the pharmaceutical company cover that from their own research budget? The CMS seems to be putting the interests of the drug company ahead of seniors.’ *(Self-identified member of the general public/taxpayer)*

Assessing the evidence overall, these commentors concluded that the benefits of aducanumab were too small or too speculative to justify the clear risks. This was exemplified by a clinician who stated:

Biogen’s trials did not show convincing evidence of cognitive improvement or quality of life, which is what I care about and what I would counsel my patients on when considering any drug intended for the treatment of Alzheimer’s dementia. I would never recommend a medication to my patients which does not have proven benefit with patient-oriented outcomes, especially given the cost and significant risks for side effects.

Such an assessment of the underlying evidence was tightly linked to criticism of the FDA’s decision to approve aducanumab as well as with thinking either that CED was appropriate or that CMS should not cover aducanumab at all.

By contrast, a smaller group of commentors expressed a belief that the existing evidence was sufficient to conclude that aducanumab offered an acceptable risk–benefit profile. Some were bullish about aducanumab’s effectiveness. For instance, one clinician described aducanumab as ‘a promising miracle drug,’ while another asserted that ‘there is a CLEAR benefit.’ More frequently, however, these commentors allowed that the evidence of efficacy was uncertain but were willing to trade some certainty for earlier access to aducanumab as ‘any modest “potential” opportunity to slow the progression of [AD] … is beneficial.’ Some of these commentors minimized the risks to patient safety, saying that brain bleeding and swelling could be ‘safely managed with proper surveillance’ or, similarly, that ‘such episodes typically resolve without sequela if detected and managed promptly and appropriately.’ Others omitted any discussion of risks or safety. Guided by their understanding of the evidence for aducanumab, most of these commentors found CED misguided because it would limit access, and a majority favored coverage. Several of these commentors also fully endorsed FDA’s approval of aducanumab.

A third group of commentors felt more evidence, and thus more research, was needed to reach any definitive conclusion regarding aducanumab’s safety and efficacy. One caregiver wrote, ‘This drug CANNOT be trusted until thorough and correct experimentation has been done on it proving that it helps either slow or reverse the effects of Alzheimer’s disease.’ Within this third group, commentors were split on CED. A majority supported CED, feeling that ‘more research is reasonable given the equipoise regarding efficacy.’ Others, however, opposed any coverage, despite wanting more data, because they felt that ‘Biogen, not taxpayers, should bear the cost of proving their drug has significant benefit that outweighs the risk.’

##### ii. Use of the Accelerated Approval Pathway

Just over 200 unique comments specifically addressed FDA’s use of the accelerated approval pathway for aducanumab. A minority of these commentors felt the agency’s use of the accelerated approval pathway was ‘appropriate under the law and good for AD patients,’ while most deemed it inappropriate. Academics and researchers as well as clinicians and other health care providers were more likely than other stakeholders to express concern about FDA’s use of the accelerated approval pathway, with ~20% of all unique comments from these stakeholder groups raising this issue.

Those opposed to use of the accelerated approval pathway voiced two main concerns. First, some noted that ‘the data from across 2 clinical trials of aducanumab showed no solid evidence that the drug has any efficacy’ on a clinical endpoint and felt that there was ‘no justification … to pretend the trial[s] did not happen.’ They felt the ‘change from endpoints measuring direct patient benefit on cognition to a surrogate endpoint late in the development process, undermines FDA’s credibility.’ Second, many felt the choice of amyloid reduction as the surrogate marker was ‘controversial.’ A physician explained why:

While the FDA has defended this decision by noting that they granted accelerated approval to aducanumab, which only requires the drug to demonstrate an effect on a surrogate marker thought to be predictive of clinical benefit, the agency has failed to note that the particularly [sic] surrogate maker underpinning aducanumab’s approval – that of beta-amyloid seen on MRI scans of the brain – has failed in over two dozen clinical trials to show any association with clinically meaningful changes in Alzheimer’s disease patients.

#### 3. The Relationship Between CMS and FDA

Commentors felt strongly about various aspects of both FDA’s and CMS’s handling of aducanumab. This colored their perceptions of the agencies; moreover, for some commentors, the agencies’ differing approaches highlighted tensions between them (Table 6).

**Table 6:** Exemplary quotes regarding FDA, CMS, and tensions between them as revealed by aducanumab

**Due to how it handled aducanumab, FDA is viewed ...**	
*… unfavorably.*	• ‘Unfortunately this decision throws into doubt the discretion of the FDA, an agency that was historically trusted to make good medical decisions.’ *(Academic (including researchers))* • ‘The FDA’s reputation has been hurt in recent years, and this decision compounds that problem.’ *(Medicare beneficiary)* • ‘This rushed approval only highlights there are serious questions as to motivation for drug approval at the FDA. This rush to approve Aduhelm did manage to underscore the unwholesome connection between the developer of the drug, Biogen, and the FDA in their after-the-fact marginalized analyses of the phase 3 trials, which had already shown the drug as ineffectual.’ *(Not specified)*
*… favorably.*	• ‘With the approval of Aduhelm, Biogen and the FDA have lit a bright candle of hope for our entire community.’ *(Person living with Alzheimer’s disease; Alzheimer’s patient advocacy organization)* • This FDA approval offered new hope for Alzheimer’s patients and their loved ones since Aduhelm is the first and only treatment that targets the pathology of the disease: amyloid beta plaques in the brain. *(Clinical society/organization)*
**Due to how it handled aducanumab, CMS is viewed …**	
*… unfavorably.*	• ‘The NCD proposed by CMS is an unprecedented and capricious effort to regulate drugs in a manner that invades the FDA’s jurisdiction, violates CMS’s own regulations and long-standing practices, and denies more than a million potential patients’ coverage to label for the first approved therapy for AD in 20 years.’ *(Alzheimer’s patient advocacy organization)* • ‘Medicare/Medicaid should absolutely continue covering this medication to treat Alzheimer’s Disease. To do otherwise is morally irresponsible and would deny folks the potential benefits being offered from this specific treatment.’ *(Not specified)*
*… favorably.*	• ‘Your decision on restricting the use of anucanumab to research and the select people who might benefit from it shows you value good science over political pressure (from the Alzheimer’s Association). I understand the frustration people have about having such limited pharmaceutical options, but a questionably beneficial drug is worse than no drug.’ *(Not specified)* • ‘Bravo for our good colleagues at CMS for denying approval of Aduhelm—and Big Pharma’s corporate patient-exploitive products whose side-effects outweigh their benefits—and for preserving best standards of evidence-based patient-centered care and practices.’ *(Clinician or other health care worker)*
**FDA and CMS are in tension because …**	
*… CMS is correcting FDA’s mistakes.*	• ‘Professionally I believe FDA’s approval of this drug was a travesty, and frankly was enormously relieved when CMS imposed appropriate constraints on Aducanumab’s use and re-imbursements.’ *(Clinician or other health care worker)* • ‘Thank you for putting some brakes on the FDA’s ill-considered approval of Aduhelm by requiring evidence Aduhelm provides clinically meaningful benefit before agreeing to pay for it.’ *(Family member/caregiver of person living with Alzheimer’s disease)*
*… CMS is undermining FDA.*	• `The draft NCD sends a signal that the CMS can effectively set aside the FDA’s approval of a new therapy and require a duplicative system for its own re-evaluation of the evidence upon which an approval is based. This proposed approach disregards the primacy of the FDA’s jurisdiction as therapy regulator, its critical role in overseeing trial design (endpoint selection, methodology, target product profile, and biomarker selection, to name a few areas), and its benefit/risk determination based on significant scientific expertise in the relevant disease state.' *(Other advocacy organization)* • ‘[T]he current proposal inappropriately undermines FDA’s Accelerated Approval pathway. This pathway enables therapies for serious, life limiting conditions to be approved and available to patients more expeditiously and relies on scientifically validated surrogate endpoints.’ *(Pharmaceutical company/industry association)*

Many commentors who viewed the FDA favorably felt that CMS had ‘overstepped its authority by essentially overruling FDA’s approval’ of aducanumab, creating ‘new evidentiary standards independent of the FDA,’ ‘usurp[ing] the FDA’s role as the exclusive arbiter of clinical data to grant or deny market access,’ and ‘essentially establish[ing] a second approval process.’ Some of these comments urged CMS not to ‘second guess the FDA’ but instead to trust the ‘scientists at the FDA [who] have reviewed the data and have decided to approve aducanumhab [sic].’

By contrast, comments that found fault with the FDA often portrayed CMS favorably—as fixing ‘the FDA’s mistake’ (ie approving aducanumab) or ‘send[ing] a signal to the FDA that accelerated approval without clinical data will not be rubber stamped for coverage.’ One patient advocacy group sympathized with CMS, writing, ‘[T]his rush [by FDA] to get drugs approved creates a dangerous and expensive risk for consumers and adds additional responsibilities on other public health agencies, such as CMS.’

Other commentors, dissatisfied with both agencies, felt the FDA was wrong to approve aducanumab and also that CMS was not doing enough to address that error. One commentor, however, cautioned that:

If the trust in the FDA has eroded, if we suspect that they are not maintaining their high standards or being influenced, not by science but by outside pressures, then we need to fix that problem. Allowing another ill-equipped agency to assume their duties is not the answer.

The commentor worried that CED would ‘open the gates for further power grabs and misuse of power.’

#### 4. Downstream Effects of the Proposed NCD on Drug Development and Evidence Generation

Nearly 150 of the unique comments explored the potential effects of CMS’s proposed use of CED on future research (Table 7). Though this theme came up relatively rarely in the comments overall, it was addressed with notable frequency by one stakeholder group: pharmaceutical companies or other industry associations. Nearly half of the 27 unique comments submitted by pharmaceutical companies or other industry associations expressed concern about the NCD’s negative downstream effects on innovation; none articulated a lack of concern.

**Table 7:** Exemplary quotes regarding downstream effects of the proposed NCD on drug development and evidence generation

**The proposed NCD would …**	
*… disincentivize Alzheimer’s disease drug development specifically.*	• ‘This decision also would apply to any future drugs that target amyloid in the brain, which is believed to play a central role in the progression of Alzheimer’s. This creates additional disincentives in an area of medical research that has waited 115 years for this first FDA-approved treatment.’ *(Other)* • ‘I think many forget that this is NOT an Aduhelm issue—this is any drug in this space to come forward. Why will any of the pharmaceutical companies want to develop these drugs to have these restrictions? They wont!’ *(Family member/caregiver of person living with Alzheimer’s disease)*
*… incentivize Alzheimer’s disease drug development specifically.*	• `Opponents of this proposed coverage decision have warned that this may have undue ramifications on future innovation of novel Alzheimer’s disease drugs. However, we view CMS’s decision as doing the opposite. … CMS’s proposed decision to cover such therapies under CED may instead send another signal to these manufacturers in incentivizing them to conduct rigorous clinical trials evaluating clinically meaningful endpoints within trial participants representative of the Alzheimer’s disease population in the premarket period, instead of confirming such benefit in post-market studies. Moreover, this decision could also communicate to manufacturers to invest into research and development of Alzheimer’s disease drugs with other mechanisms of action with strong and clear associations with cognition and function.' *(Clinician or other healthcare worker; Academic (including researchers))*
*… disincentivize drug development generally.*	• ‘Perhaps most concerning, in the long term to be sure, is that CMS is sending a strong signal to the biotech investment and innovation market that FDA scientific determinations are now subject to CMS second-guessing. This proposal, from an investor and innovator standpoint, is profoundly anti-innovation and anti-patient.’ *(Other)* • `The uncertainty that is created by the potential for a CMS reassessment of a biomarker, undermining FDA’s determination and potentially creating restrictions to a product for the population for which it is indicated, would act as a significant disincentive to research.' *(Pharmaceutical company/industry association)*
*… incentivize companies to collect evidence their drugs work.*	• `I strongly support – and urge CMS to maintain – the decision NOT to pay for Aduhelm except in the setting of clinical trials. Paying for coverage of a drug that has no demonstrated evidence of efficacy (but demonstrated evidence of harm) will not only hurt patients now (ie those who would receive the drug outside the setting of clinical trials), it would set a dangerous precedent. … If pharmaceutical companies (and the patient advocacy groups they bankroll) know that the evidentiary bar for coverage is so low (or non-existent), they will not have the incentive to develop (and support) treatments that actually work (rather than those that can just get coverage).' *(Not specified)* • `We write in support of the proposed “coverage with evidence development” (CED) approach … Consumers’ interests are not served by rewarding drug manufacturers for a drug with weak evidence for effectiveness.' *(Other advocacy organization)*

Nearly all comments on this theme, not limited to those submitted by pharmaceutical companies or other industry associations, expressed the view that CED would negatively—perhaps even catastrophically—affect drug development going forward. Approximately one-third of these commentors focused narrowly on AD and argued the proposed NCD would ‘stifle innovation and investment in AD research’ or, similarly, ‘greatly hamper ongoing and future Alzheimer’s research, creating a chilling effect delaying and disincentivizing badly-needed scientific innovation to better treat and ultimately prevent this currently terminal and progressive disease.’ ‘[B]ig Pharma,’ they warned, ‘[won’t] continue to invest money in developing a treatments [sic] that will have no future in reaching the market.’ A clinician wrote, ‘If your hope was to hamstring and disincentivize pharmacotherapy development for the millions of patients and families suffering from AD, congratulations [CMS]. You have achieved your misbegotten aim.’

Other commentors expressed concern that the effects of the proposed NCD on drug development would be felt beyond AD, adversely affecting patients with other serious conditions. They accused CMS of setting an ‘alarming precedent’ by not covering ‘a treatment approved by the Food and Drug Administration.’ Trial sponsors, they explained, need to be able to ‘rely on FDA’s determinations on safety and efficacy.’ If CMS denied full coverage to aducanumab, these commentors warned, it would have the effect of ‘stifl[ing] innovation and discourag[ing] biopharmaceutical companies from investing in the riskiest research and development of treatments for serious diseases.’ An advocacy organization argued that CED was ‘bad public policy’ that would ‘disproportionately impact individuals with rare and ultra-rare conditions and deter development of new therapies.’ The view that CED would discourage pharmaceutical companies from investing in research was closely linked to the view that CMS was encroaching on the FDA, discussed in §IV.A.3.

Some commentors voiced worries that CED would ‘discourage utilization of [the] Accelerated Approval pathway.’ A frontotemporal dementia advocacy organization explained that by requiring additional trials for evidence development, CMS was ‘undermining the FDA’s use of the accelerated approval pathway.’ Meanwhile, a pharmaceutical company wrote that ‘while CMS does not explicitly call into question the use of the Accelerated Approval pathway, we note the importance for manufacturers and FDA to identify surrogate biomarkers that are appropriate to speed development of new therapies and access for patients with high-unmet need diseases.’

Commentors worried about the downstream effects of CED generally rejected it, with roughly half seeking full coverage as the alternative to CED. Approximately 1 in 10 sought to mitigate the effects by asking that CED apply narrowly to aducanumab rather than to all drugs in the class.

Just a handful of commentors felt CED would favorably influence future drug development, whether for AD or other indications. Of these, many shared a belief that requiring CED would signal to pharmaceutical companies that they should ‘invest into research and development of … drugs with strong and clear’ signs of efficacy and ‘conduct rigorous clinical trials evaluating clinically meaningful endpoints … in the premarket period, instead of confirming such benefit in postmarket studies.’ Some commentors also felt CED would ‘support the development of future antiamyloid [sic] mAbs by not discouraging clinical trial participation’ and would ‘allow the field to gather badly needed phase 3 data on other anti-amyloid antibodies like donanemab and lecanemab.’

#### 5. Downstream Effects of the Proposed NCD on Health Equity

As noted above, commentors widely acknowledged that AD is characterized by disparities. Nearly 300 unique comments addressed how CED might affect disparities (Table 8). Academics, researchers, clinicians, and other health care workers invoked health equity considerations more frequently than other stakeholders.

**Table 8:** Exemplary quotes regarding downstream effects of the proposed NCD on health disparities

**The proposed NCD would …**	
*… harm disadvantaged populations.*	• ‘And now, once again, we have raised hopes for an Alzheimer’s disease modifying therapy only to now declare that it will be available primarily to affluent, white Americans, not the communities who have the greatest need, the communities whose trust and trial participation we seek to encourage.’ *(Person living with Alzheimer’s disease; Alzheimer’s patient advocacy organization)* • ‘The concentration of CED trials at major academic medical centers engendered by the agency’s proposal could worsen existing disparities in Alzheimer’s treatment against minority racial and ethnic groups, socioeconomically disadvantaged Americans, and persons residing in rural areas.’ *(Pharmaceutical company/industry association)* • ‘But with CMS’s proposed decision limiting coverage to those in clinical trials, access is severely restricted to only those few who live near research institutions, or can afford to pay out-of-pocket.’ *(Alzheimer’s patient advocacy organization)*
*… help disadvantaged populations.*	• ‘We support the requirement that the RCTs reflect “the diversity of patients [and be a] representative [cross-section] of the national population diagnosed with [Alzheimer’s],” (eg age, gender, disease severity). … This expansion of trial populations will proactively avoid potential disparities in care.’ *(Pharmaceutical company/industry association)* • `We strongly support CMS’ emphasis on the importance of evaluating the safety and effectiveness of treatment on different groups beyond those enrolled in the clinical trials, including minorities, underserved, and low-income individuals, many of whom are at greater risk for developing AD and who may be more likely to have a missed diagnosis of the disease. Without this additional research, it will be unclear whether access to this treatment, with its accompanying risks, is in the best interests of diverse populations that have been systematically underserved by prior clinical trials.' *(Insurer or other payer)*
… *favor those who can afford to pay out of pocket.*	• ‘Restricting access only to patients that can afford to pay out of pocket is unfair and worsens the inequity in health care.’ *(Not specified)* • ‘The draft NCD establishes two distinctly separate tiers of access. Wealthy individuals who qualify for the drug can pay out of pocket. The rest of those who may benefit from such a treatment can only access the drug through clinical trial participation.’ *(Other advocacy organization)*
*… discriminate against AD patients as a group by treating them differently than patients with other diagnoses.*	• ‘This proposed decision is inequitable compared to decisions made in the past for other terminal diseases without any treatments. When the first treatments for HIV or various cancers were approved by the FDA, they were all covered fully by CMS. It is not fair to treat Alzheimer’s Disease treatments any differently.’ *(Family member/caregiver of person living with Alzheimer’s disease)* • ‘[T]his seems highly discriminatory against a very specific population—those with a diagnosis of Alzheimer. I guess they should have just had cancer instead—that’s the message you are sending.’ *(Family member/caregiver of person living with Alzheimer’s disease)* • ‘If this were a drug for children or young adults there would no such roadblock—CMS is clearly ageist.’ *(Clinician or other health care worker)*

Worries about inequitable access to the kinds of trials outlined in the proposed NCD, and therefore to aducanumab, predominated and spoke to multiple (potentially intersecting) axes of disadvantage. Commentors cautioned that CED would hit ‘the underserved (blacks, Hispanics, Native Americans, women, etc.) the hardest.’ One stated, ‘[W]e do know that women, Blacks and Hispanics are disproportionately impacted by the disease,’ before asking, ‘Why then does the approach proposed by CMS make access to treatment available only to those with access to research institutions?’ Another commentor accused CMS of ‘practicing big city bigotry’ because CED would have the effect of ‘concentrating access to anti-amyloid mAbs [sic], for the foreseeable future, among people who live in major metropolitan areas or who have the resources to frequently travel to those areas.’ Some commentors also felt that ‘the restriction on coverage to clinical trial sites will acutely impact disadvantaged and socioeconomically deprived communities, as identification, enrollment and participation in a clinical trial will likely require significant resources.’ More broadly, several commentors were concerned that CED would lead to two tiers of patients, those who could simply pay and those who would have to participate in research to access aducanumab. Commentors worried about access to trials favored full coverage to CED at a rate of nearly 3 to 1.

Some commentors focused on CMS’s proposal to require representative research populations, seeing it as a tool to advance health equity. One commentor heralded an ‘unprecedented opportunity to shed light on a number of overlapping and embedded barriers to understanding the nature of dementia risk.’ Another noted that representative research samples could reduce the challenge of generalizing ‘trial results from a more homogenous population’ to AD patients broadly. Other commentors, though, expressed tempered optimism. A neurologist acknowledged the ‘absolute need for studies that are more fully representative of the US population’ but warned that the requirement to conduct RCTs ‘mandate[s] the kind of protocols that have proved insensitive to the needs of diverse communities and unable to engage representative samples.’ Echoing this, another commentor observed that populations ‘disproportionately affected by AD are also more likely to have comorbidities’ and thus to be ineligible to participate in the kinds of trials envisioned by the proposed NCD; this commentor concluded that CED had the potential to ‘worsen disparities despite CMS’s goal of patient representativeness.’

As just discussed, many of the comments focused on disparities within the pool of AD patients. But nearly 100 unique comments focused on disparities between people living with AD and people living with other serious conditions. Comparisons were most frequently drawn to patients with diagnoses of cancer or HIV, who were perceived to be similarly situated to AD patients but treated more favorably by CMS. One commentor asked, for example, ‘[I]f CMS can cover other diseases like cancer and HIV, why can’t it cover AD?’ Such points were most often made by family members and caregivers of people with AD and clinicians and other health care providers. Commentors who worried about disparate treatment of AD patients most often favored full coverage.

#### 6. Aducanumab’s Cost, Value, and Affordability

Just under one-third of the unique comments discussed aducanumab’s cost, value, or affordability (Table 9). Many commentors – most often self-identified Medicare beneficiaries, family members and caregivers of people with AD, and clinicians and other health care providers – pointed out how expensive aducanumab was. While some spoke of ‘a breathtakingly steep $56,000 price tag,’ that is, aducanumab’s initial price, others noted that ‘the initial price … was halved to $28,000 per person for a year of treatment.’ Among the unique commentors, more than 70 described aducanumab’s price as ‘outrageous.’

**Table 9:** Exemplary quotes regarding aducanumab’s cost, value, and affordability

**The cost of aducanumab …**	
*… is not justified by the current evidence on safety and efficacy.*	• ‘I am totally against Medicare accepting this so-called Alzheimer’s drug at its ridiculously high price. Has it even been proven effective? Medicare, do not accept this drug until it has been proven to be effective and the price is greatly lowered. Common sense is needed.’ *(Not specified)* • ‘Not only is there no evidence of benefit, but the drug poses significant risks and is outrageously expensive. CMS must hold the line and pay for drugs only when clear evidence of their efficacy and safety has been established.’ *(Academic (including researchers))*
*… may be justified.*	• ‘Antibody therapies represent a way to avert skyrocketing Medicare costs resulting from increased AD prevalence in our aging population. In summary, this penny wise and dollar foolish implementation creates more problems than it solves.’ *(Academic (including researchers))* • ‘As for the costs of such drugs, it may well be that the up-front cost of monoclonal antibody treatment is offset by the current long-term costs of the pre-existing disease management, while improving the quality of life for the afflicted and their caregivers.’ *(Clinician or other health care worker)*
**Paying for aducanumab …**	
*… could bankrupt Medicare.*	• ‘Please don’t let big pharma bankrupt Medicare over the new Alzheimer’s drug. Please limit coverage to the test group, Don’t force Medicare to pay for the drug (at over $50,000.00!!!) for all seniors interested.’ *(Not specified)* • ‘Besides putting patients at risk for severe side effects for no proven benefit, it could bankrupt our Medicare system.’ *(Family member/caregiver of person living with Alzheimer’s disease)*
*…* *will use scarce Medicare resources that could better be used elsewhere.*	• ‘I do not believe it should be offered, especially because its exorbitant cost will cause real hardship and weaken Medicare’s ability to protect the elderly from diseases drugs can actually help with.’ *(Not specified)* • ‘Aduhelm is incredibly costly … this same taxpayer funding could be used to provide evidence-based non-pharmacological treatments that improve the lives of persons living with dementia and their caregivers.’ *(Academic (including researchers))*
***…*** *will increase Medicare premiums and harm beneficiaries.*	• ‘The research I read states that it is usually not helpful in stopping Alzheimer’s and that it is outrageously expensive. So expensive that it will drive up the cost of health insurance for all seniors. We seniors can not afford to have this drug covered under Medicare.’ *(Medicare beneficiary)* • `[M]y monthly Medicare D costs increased 21% from $140 to $170 for 2022. This was a noticeable increase in my budget. The number of Medicare enrollees who will eventually be prescribed this medication, which is produced by Biogen, is unknown. Though with more of the boomer generation and those following generations reaching the age when dementia and Alzheimer’s is diagnosed, the number of prescriptions is likely to skyrocket. Then Medicare costs further increase, something I can not afford since I live on a fixed income. I submit that almost everyone dependent on Medicare for medical and prescriptions will not maintain their standard of living if Medicare costs skyrocket.' *(Medicare beneficiary)*

Commentors were not merely upset by aducanumab’s absolute cost. Many made nuanced comments about aducanumab’s value—that is, its perceived utility or worth. As reflected by the comment that aducanumab’s ‘cost is not justified by effectiveness,’ ideas of value were closely linked to commentors’ evaluation of the evidence of safety and efficacy, discussed in § IV.A.2.i above. People who deemed aducanumab a low- or no-value drug correspondingly expressed low willingness to pay (ie the maximum amount they would pay to receive the drug). For example, a commentor described herself as ‘outraged by the idea’ of paying for a drug that ‘costs too much and doesn’t work.’ These commentors generally approved of CED. Some, though, felt CMS had not gone far enough and should instead ‘hold the line and pay for drugs only when clear evidence of their efficacy and safety has been established.’

Select commentors, who viewed the evidence of safety and efficacy favorably, suggested that the aducanumab’s cost might be justified if one considered the benefits to patients and caregivers as well as the savings that would accrue as a result of reduced spending on dementia care. One, for instance, asserted, ‘The cost of this drug is FAR less compared to the cost of long-term [sic] care for those Americans’ with AD. Consistent with this view, these individuals regarded CED as ‘a step backward’ that would ‘affect patients’ welfare detrimentally.’

##### i. Effect on Medicare and Medicaid

Many of the unique comments addressing cost, value, or affordability noted the ‘massive financial burden to society’—particularly to Medicare—of paying for an expensive treatment like aducanumab with a ‘zillion alz [sic] patients … clamoring for its use.’ A comment from the Medicare Payment Advisory Commission (MEDPAC), a nonpartisan independent legislative branch agency that provides Congress advice on the Medicare program,^89^ explained that Biogen ‘has stated that the product is appropriate for between one million to two million individuals.’ The letter went on:

At the current price of $28,200 a year of maintenance therapy, Medicare Part B FFS [fee-for-service] spending and beneficiary cost sharing could total $1.5 billion annually if 50,000 FFS beneficiaries received the product and $15 billion annually if 500,000 FFS beneficiaries received the product. Thus, with substantial uptake, the product has the potential to swamp current Part B drug spending.

Affordability, or a measure of cost relative to what the purchaser is in fact able to pay, also came up in the comments. A handful of commentors worried that Medicare could ‘be bankrupted’ by aducanumab. One even stated that ‘we could foresee not only bankruptcy of Medicare ... but of whole federal govt ... for very tenuous claim [sic].’ Commentors concerned about the impact of aducanumab on Medicare spending tended either to favor CED as a means of ‘stewardship’—that is, containing the financial impact of aducanumab and other drugs in the class—or to oppose any coverage whatsoever.

Some comments, often submitted by self-identified Medicare beneficiaries, highlighted the effects of aducanumab on Medicare premiums. Most correctly specified that ‘Part B premiums are increasing.’ Several mistakenly indicated that Part D premiums would increase, saying for instance that paying for aducanumab would cause ‘Part D premiums to skyrocket’ or that ‘[r]aising Medicare Part D premiums is totally unfair.’ Judgments on aducanumab’s value were woven into these comments. ‘It’s not fair to other Medicare clients (like me!),’ one commentor said, ‘to pay more for our premiums for this one new drug that is not scientifically proven to help.’ Further, commentors voiced concern that Medicare’s ‘inordinately high premium increase’ would ‘cause tremendous hardship’ for senior citizens living on fixed incomes. Contextual factors like living in ‘a time of high inflation’ heightened this concern. ‘Cost[s] of housing, food and other essentials continue to rise – along with our Medicare premiums,’ one explained before adding that this was ‘plummeting many of us further into poverty each year.’

Some of the comments made broad statements that neither Medicare nor Medicaid should pay for aducanumab. Yet, the effect of aducanumab on Medicaid spending went largely unaddressed. When it was addressed, it was primarily addressed by organizations. The National Association of Medicaid Directors (NAMD) submitted a comment that recognized ‘the sound clinical reasoning behind’ CED but noted that ‘limited Medicare coverage will shift primary coverage responsibility onto state Medicaid programs for individuals dually eligible for both Medicare and Medicaid who are prescribed these therapies.’ This is because, as explained in a comment submitted by Medicaid Health Plans of America, the ‘Medicaid Drug Rebate Program (MDRP) requires that participating pharmaceutical manufacturers provide specific discounts to state Medicaid Programs in exchange for coverage of the manufacturer’s FDA-approved products.’ NAMD argued that ‘Medicaid programs should not be expected to provide widespread coverage for therapies that do not meet evidentiary standards for similarly widespread Medicare coverage.’ The Pharmaceutical Care Management Association called on CMS to ‘ensure a consistent application of coverage guidance across its programs,’ while the Blue Cross Blue Shield Association requested additional guidance for state Medicaid directors on ‘guardrails they may put in place to adequately protect patients’ and argued that state Medicaid programs should have the flexibility to cover only proven therapies.

##### ii. Opportunity Costs

Several commentors noted that paying for aducanumab would have ‘“opportunity costs” – in other words, the impact of diverting governmental … resources to cover [aducanumab].’ For instance, one commentor exclaimed, ‘The drug’s high price will improperly siphon taxpayer funds away from better medicine for taxpayers!’

Some commentors, however, realized funds going to aducanumab could not easily be redirected to improve dementia care. For instance, two researchers who had conducted a public opinion survey on aducanumab reported in their joint comment that:

Greater than 60% [of our survey respondents] agreed that the money Medicare would spend on aducanumab would be better spent elsewhere, such as long-term care for Medicare beneficiaries who need it, whether or not they have Alzheimer’s, or on other effective care for Medicare beneficiaries. … [O]ur finding that the public is willing to make tradeoffs between spending on aducanumab and other clinical or social services is notable given that Medicare does not currently cover social services like homemaker services, such as shopping and cleaning, that may benefit those with mild Alzheimer’s disease or dementia and their caregivers.

Other comments provided anecdotal evidence consistent with these survey findings saying, for example: ‘There are ... [s]o many services people with dementia – whatever the cause – need today to live with dignity and to spare their families emotional and financial burn-out. Medicare does not pay for what we need.’

##### iii. Pharmaceutical Industry Greed

More than 10% of the unique comments characterized Biogen specifically or the pharmaceutical industry broadly as greedy and immoral (Table 10). As an example of the former, an academic wrote that ‘profits over people is a cruel mindset that this company [Biogen] is inflicting on families in immeasurable pain caused by Alzheimer’s Disease.’ As an example of the latter, a clinician wrote, ‘Big Pharma’s greed is unsurpassed.’ Commentors argued that ‘greedy Big Pharma’ charges CMS unreasonable prices for drugs like aducanumab—prices that harm patients but also adversely affect ‘tax paying senior citizens and younger still-employed citizens who contribute to the social safety net with their hard earned dollars.’ Suggesting a widespread distaste for pharmaceutical companies, such comments were submitted by a diverse group of stakeholders that included self-identified Medicare beneficiaries, family members and caregivers of people with AD, and clinicians and other health care providers. These commentors often saw CED as a means of preventing ‘Medicare’s funds/coffers [from] be[ing] “legally” robbed.’

**Table 10:** Exemplary quotes regarding pharmaceutical industry greed and price negotiation

**Pharmaceutical companies are greedy …**	
*… and seeking to profit at the expense of patients.*	• ‘I say this as a 77 yo, because I live in the United States, and have spent my life watching Big Pharma run roughshod over ordinary folks to get their drugs approved, on the market, and collect their huge monies.’ *(Medicare beneficiary)* • ‘[M]illions of Medicare recipients will pay for this gross display of greed from which we will receive no benefit at all.’ *(Medicare beneficiary)*
*… and seeking to profit at the expense of taxpayers.*	• `The hubris of any drug company complaining that their marginally proven drug will not be granted unfettered access to the millions of taxpayers covered by Medicare is pretty outrageous. The fact that at the first sign of pushback the cost was lowered is a very telling sign. … They should not be allowed to make profits for their shareholders with a marginal product that is being subsidized by taxpayers.' *(Self-identified member of the general public/taxpayer)* • ‘AND I AM TOLD THAT A LOT OF THE MONEY THESE COMPANIES SPEND ON RESEARCH COME FROM TAX PAYER FUNDS. THEY ARE MAKING RECORD BREAKING PROFITS, PAYING THEIR EXECUTIVES OUT OF SIGHT SALARIES AND BONUSES. IN OTHER WORDS THE TAX PAYER IS GETTING SCREWED ROYALLY.’ *(Not specified)*
*… and Congress is too conflicted to address it.*	• ‘[G]iven the influence of the pharmaceutical lobby on Congress, we are standing on the proverbial slippery slope and the interests of the public/taxpayer must be protected by some entity that will address the situation objectively.’ *(Self-identified member of the general public/taxpayer)* • `It is inconceivable that the US Congress cannot agree to allow Medicare to negotiate drug prices. ... It is nothing short of CORRUPTION that the pharmaceutical companies are lining the pockets of our legislators, elected to represent the American people—legislators on BOTH sides of the aisle that are enriching themselves while ignoring the cost to Americans and to taxpayers. It is time for change. It is BEYOND time for change. Democrats and Republicans need to finally after decades of looking out for themselves...do what they were elected to do!!! Represent the EVERYDAY people of this country!!!' *(Not specified)*
*… and CMS should be able to address this by negotiating prices.*	• ‘Medicare must be able to negotiate the cost of all drugs to prevent corporate greed.’ *(Not specified)* • ‘There is no excuse for their greed to inflate the profits made on drugs and treatments without the largest health care plans (Medicare and Medicaid) being allowed to negotiate the costs that must eventually be passed on to every US citizen through taxes.’ *(Not specified)*

##### iv. Price Negotiation

Several unique comments included the observation that CMS is a price taker and cannot negotiate drug prices to fairly reflect a drug’s value (Table 10). As a result, though aducanumab ‘may not be effective at preventing or treating Alzheimer’s, … Medicare would be on the hook for tens of thousands of dollars per patient with no way to negotiate a lower price.’

Some of these comments included a general point that CMS ought to be able to combat high drug prices through negotiation because ‘accepting the cost they [pharmaceutical companies] put forward is undemocratic and*—*crazy.’ One clinician observed, ‘The United States is socially and politically behind most developed countries because of a process that does not allow Medicare to negotiate with Pharma.’ A number of commentors spoke to the consequences of this prohibition. ‘It is unconscionable,’ one explained, ‘that Americans are paying more than people throughout the globe for the drugs they need.’ Another asked, ‘WHY are Americans being overcharged and forced to choose between paying for food or heat OR an RX?’ Several comments suggested that the cost of CMS’s inability to negotiate could be measured not just in dollars but in lives. One commentor wrote:

U.S. citizens are subsidizing pharmaceutical companies because we are the only country that cannot negotiate with them. Paying the highest price for medications is causing great harm to the health and welfare of U.S citizens. The increase[d] price for insulin and epi pens has actually killed U.S. citizens due to their not being able to afford the needed amount of the medication.

A few commentors who viewed the pharmaceutical industry as greedy also viewed Congress as too conflicted to do ‘their job for the taxpayers’ and address high drug prices. These commentors felt that representatives ‘on “both sides of the aisle”’ take ‘bribes aka large donations’ and ‘money from the pharma lobby.’ As a result, ‘Big Pharma has purchased enough Congressmen and Senators to allow them to do this.’ One commentor, who described the pharmaceutical industry as ‘protected by lobbyists paying off congress members,’ asked, ‘Where are the ethical people in Congress and in the government?’ before imploring, ‘Please find a conscience and let the government negotiate for Medicare med prices.’

#### 7. Hope and False Hope

The nearly 300 commentors who addressed hope (Table 11) were nearly evenly divided on whether aducanumab provided genuine hope to Alzheimer’s patients and their families or promoted false hope. Family members or caregivers for persons with AD were found on both sides of this debate, invoking both hope and false hope more frequently than other stakeholders.

**Table 11:** Exemplary quotes regarding hope and false hope

**Aducanumab offers patients and caregivers …**	
*… hope.*	• ‘Although it may not help, at least aducanumab has given us a glimmer of hope in fighting this terrible disease.’ *(Family member/caregiver of person living with Alzheimer’s disease)* • ‘Even though there are potential serious side effects, the drug offers hope for patients who otherwise will experience the progression of Alzheimer’s disease. As the care giver for my [PHI Redacted] who had Alzheimer’s disease, I can say that without a doubt that the potential for the medication to stop the ravages of Alzheimer’s disease far outweighs any possibility of side effects.’ *(Family member/caregiver of person living with Alzheimer’s disease)* • ‘As the field makes its way into the devastating disease of AD, it is my sincere wish that those suffering are provided the hope to preserve their precious minds that they yearn for. There has never been more hope for AD treatment than there is now. Why does CMS want to strip patients of that?’ (*Clinician or other health care worker)*
*… false hope.*	• ‘Without any evidence that this drug actually works, it is snake oil, and taxing to patients who cling to hope but also need to spend their remaining time wisely.’ (*Clinician or other health care worker)* • ‘People with Alzheimer’s aren’t marks—they are human beings who deserve to be treated with dignity and respect—not sold false hope.’ *(Family member/caregiver of person living with Alzheimer’s disease; Self-identified member of the general public/taxpayer)* • ‘Their profits should not be at the expense of the rest of us, certainly not those on Medicare and with Alzheimer’s, whose desperation is being exploited.’ *(Not specified)*
*… a choice.*	• ‘One concern cited about Aduhelm is potential side effects: headache (most common), diarrhea and confusion or (less common) falls and brain swelling/bleeding. People living with Alzheimer’s should have the right to weigh these risks in consultation with their physicians. Since the disease leads to certain death, most would likely consider potential side effects worth the risk.’ *(Other)* • `It is hard for me to understand why this drug would be kept from people suffering from this cruel disease. All drugs have side effects. These are trade off’s that people deserve the opportunity to make informed decisions about.' *(Family member/caregiver of person living with Alzheimer’s disease)* • ‘The draft memorandum limits the autonomy of those living with dementia or Alzheimer’s disease to make informed decisions about their own care and whether they themselves would be willing to assume any risk in order to potentially benefit from receiving treatment.’ *(Alzheimer’s patient advocacy organization)*

Commentors who described aducanumab as offering hope often emphasized that AD is ‘a disease with no available cure’ or that AD patients are ‘waiting for a treatment to save their lives.’ Among these commentors, some expressed optimism about aducanumab’s effects, stating, for instance, that it might ‘slow down the progression of a disease that steals so much from families.’ Commentors did not, however, necessarily need to believe aducanumab worked to feel it offered hope. Some were agnostic or even skeptical about the drug’s efficacy but felt that something was better than nothing. Exemplifying this, one commentor urged that ‘[t]he uncertainty in long-term effectiveness … be balanced against … the devastation that is certain without the treatment.’ This commentor concluded, ‘Hope has value even when the treatment ultimately falls short.’ Generally, commentors who linked aducanumab to hope rejected CED, preferring full coverage, because it was ‘cruel to deny people the opportunity to seek that hope.’

Conversely, other commentors worried aducanumab offers ‘false hope … packaged up as an effective treatment’ or cautioned that it is ‘dangerous to give patients and their caretakers false hope on a drug that has shown virtually no clinical benefit’ but has clear risks. They also warned that ‘peddling an ineffective medicine (if you can call it that because medicine by definition has to be effective) is … very dangerous as it gives patients false hope [and] stopes [sic] them from seeking other effective alternatives.’ Most of these commentors felt FDA should not have approved aducanumab (§ IV.A.2). Additionally, several linked false hope to corporate greed (§ IV.A.6.iii), asserting for example that ‘this episode is the epitom[e] of a scam on desperate hopless [sic] people’ or that aducanumab ‘exploits the hope [of] patients and their families.’ Generally, individuals concerned about false hope either supported CED or felt CMS should not cover aducanumab at all, suggesting that offering broader coverage would make CMS ‘complicit in providing families and patients false hope.’

#### 8. Freedom to Make a Personal Choice

Approximately 60 commentors suggested that patients should have the opportunity to weigh the benefits and risks of aducanumab in consultation with their health care provider and decide for themselves whether to take it (Table 11). While many took no firm position on CED, half favored full coverage over CED. Clinicians in particular worried that, by proposing CED, ‘CMS has effectively removed the decision from the physician and his or her patient … [which] can be interpreted as a violation of physician-patient autonomy to make the best treatment determination.’ Clinicians and other health care workers were not alone in invoking autonomy-based arguments but were more likely than other stakeholders to do so.

### IV.B. Duplicate Comments

We identified 6456 duplicate comments, reflecting 18 different ‘parent’ comments. The frequency of duplicates associated with a given parent comment ranged from a minimum of 10 to over 5000.^90^ Thirteen parent comments were duplicated fewer than 30 times. Most commentors submitting duplicates did not self-identify as belonging to any stakeholder group; of those who did, 609 identified as a family member or caregiver for a person living with Down Syndrome, 193 identified as clinicians, and roughly equal numbers identified as Medicare beneficiaries (142) or as a family member or caregiver for a person living with AD (126).

Duplicate comments generally consisted of just a few sentences or paragraphs. The basic language was provided, as discussed below, by a sponsoring organization or organizations seeking to mobilize like-minded individuals. Minor changes included substitution of one word for another. About 1 in five commentators added additional personal details to the basic text, such as where they lived or how long they had been a caregiver for a person living with dementia, elaborated on ideas from the parent comment, or included additional perspectives.

Many of the themes we developed in § IV.A, when presenting the results of our analysis of unique comments above, were also evident in the duplicate comments. There were, however, at least two notable differences. First, reflecting their shared origin, the duplicates had a directional consistency the unique comments lacked. For example, both the unique and duplicate comments addressed FDA’s approval of aducanumab. But whereas there was some heterogeneity in how unique commentors understood the adequacy of the evidence undergirding aducanumab’s approval (§ IV.A.2.i), the overwhelming number of duplicate commentors deemed the evidence lacking. Second, some topics were far more prominent in one set of comments than the other. Duplicate commentors touched on FDA’s use of the accelerated approval pathway with far greater frequency than unique commentors, at a ratio of roughly 25 duplicates for every one unique comment (§ IV.A.2.ii).

#### 1. Most Common Duplicates

The four most common parent comments (Table 12) each appeared more than 250 times.^91^ The duplicate comment that we dubbed ‘Galling Disregard for Science’ was submitted by more than 5000 commentors and criticized FDA’s decision to approve aducanumab, asserting that the agency’s credibility was damaged as a result. It called on CMS not to compound FDA’s error but to ‘issue a national coverage determination that excludes Aduhelm from coverage under the Medicare program.’

**Table 12:** Common language from the four most common duplicates

**Galling Disregard for Science**
'The Food and Drug Administration’s decision to approve Aduhelm for treatment of Alzheimer’s disease showed a galling disregard for science and eviscerated the agency’s standards for approving new drugs. Because of this reckless action, the agency’s credibility has been irreparably damaged. The approval of Aduhelm was based on seriously flawed post hoc analyses of two identical phase 3 trials that were stopped early because a preliminary review of the data found that the trials, if continued to completion, were unlikely to show the drug benefitted Alzheimer’s disease patients. Moreover, the integrity of the FDA’s review of the marketing application for Aduhelm was dangerously corrupted by the unprecedented and inappropriately close collaboration between Biogen and the FDA during the analyses of data from the key clinical trials of the drug after the termination of the phase 3 clinical trials because of futility.
CMS must not compound the FDA’s egregious error in approving Aduhelm on June 7, 2021. Given the lack of scientific evidence that Aduhelm provides any clinically meaningful benefit in terms of cognitive function outcomes in Alzheimer’s disease patients, the drug cannot possibly be deemed reasonable and necessary for treatment of such patients. I urge CMS to issue a national coverage determination that excludes Aduhelm from coverage under the Medicare program. It’s imperative to put patients and consumers before Big Pharma profits.'
**Discriminates Against Down Syndrome**
'I have recently become aware of a new Alzheimer’s drug called aducanumab and that CMS has proposed coverage that excludes people with Down syndrome and other intellectual and developmental disabilities. I understand the need to make sure treatments are safe, but this is the wrong path forward. CMS must abandon the proposed CED process because it discriminates against people with intellectual and developmental disabilities now and into the future. Having Down syndrome should not prevent a patient from accessing Alzheimer’s treatments. If one patient covered by Medicare or Medicaid can access an Alzheimer’s treatment, every patient covered by Medicare or Medicaid should be able to if their doctor believes it’s right for them.'
**Same Access for Down Syndrome**
'We know that [our family member is] more likely than other people to develop Alzheimer’s disease, so even though there’s no cure now, it’s very important to me that [they have] access to any treatments that will be developed in the future.
I’ve become aware that the Centers for Medicare & Medicaid Services might exclude [them] from clinical trials related to new Alzheimer’s treatments. I strongly believe that CMS should not move forward with any coverage process that excludes people with Down syndrome and other disabilities. If people on Medicare or Medicaid without disabilities can access new treatments, people with disabilities who are covered should have the same access.”
**Health Equity for Down Syndrome**
'Having Down syndrome should not prevent a patient from accessing Alzheimer’s treatments. As a matter of health equity, if CMS covers Alzheimer’s treatments for any Medicare/Medicaid recipients, then it must cover these treatments for all of them. When a physician determines that a treatment is right for a covered patient, then that patient should have access regardless of race, ethnicity, religion, income, geography, gender identity, sexual orientation, or disability.'

The three other most common duplicate types were all related to the proposed exclusion of individuals with Down Syndrome or other IDD from coverage criteria. ‘Discriminates Against Down Syndrome’ appeared just over 500 times. This duplicate called on CMS to ‘abandon the proposed CED process’ due to concerns that it prevented people with Down Syndrome from accessing mAbs directed against amyloid for treatment of AD; further, it argued that these individuals should enjoy access comparable to that of other Medicare and Medicaid recipients. Appearing around 300 times, ‘Same Access for Down Syndrome’ emphasized the high burden of AD for people with Down Syndrome and argued that CMS should not exclude them from trials but instead grant them the same access to Alzheimer’s treatments enjoyed by others. Finally, ‘Health Equity for Down Syndrome’ appeared over 250 times. It focused on the importance of covering Alzheimer’s treatments for all patients if their physician determines treatment is right for them. Many comments coded as duplicates included text from two or more parent comments.

#### 2. Origins of Parent Text for the Most Common Duplicates

Because duplicates reflect the efforts of organizations to mobilize like-minded individuals, it is important to understand their origins. The text that formed the basis of the ‘Galling Disregard for Science’ comment apparently originated with Public Citizen, a nonprofit consumer advocacy organization with a self-described mission to ‘defend democracy, resist corporate power, and fight to ensure that government works for the people – not big corporations.’^92^ On June 7, 2021, the day FDA granted accelerated approval to aducanumab, the director of Public Citizen’s Health Research Group Dr Michael Carome released a statement that began:

The FDA’s decision shows a stunning disregard for science and eviscerates the agency’s standards for approving new drugs. Because of this reckless action, the agency’s credibility has been irreparably damaged. … The close collaboration between the FDA and Biogen before and after the submission of the company’s marketing application for aducanumab dangerously compromised the integrity of the agency’s review and culminated in a biased agency assessment of the drug.^93^

Carome called for an investigation into the interactions between FDA and Biogen before concluding:

Approving aducanumab despite the lack of evidence of effectiveness plus the well-documented risk of serious harm will raise false hope for millions of Alzheimer’s patients and their families, potentially bankrupt the Medicare program because of the drug’s projected exorbitant price and impede for years the development of other experimental treatments for the disease.^94^

On August 11, 2021, Public Citizen released comments specifically addressing the proposed NCD.^95^ The brief introduction to the longer document incorporated portions of Carome’s June 7 statement with only minor modifications while also building on several of his points.^96^ Additionally, it admonished that ‘CMS must not compound the FDA’s egregious error in approving aducanumab’ and called for the exclusion of aducanumab from coverage under the Medicare program.

Near the end of the CMS public comment period, on February 8, 2022, Social Security Works—a social welfare organization that ‘fight[s] to protect and expand Social Security, Medicare, and Medicaid; lower drug prices; ensure economic justice for all; and guarantee health care as a human right’^97^ —posted detailed instructions for submitting comments to CMS on its website.^98^ The post included a statement (ie the parent comment) for people to submit to CMS. The text of this statement, though not attributed to Public Citizen, is taken nearly verbatim from the introduction to Public Citizen’s August 11 comments, just described. Minor edits included the substitution of the brand name, Aduhelm, where Public Citizen had used aducanumab, as well as changing ‘Public Citizen therefore urges CMS’ to ‘I urge CMS.’^99^ The most substantive change was adding a concluding sentence: ‘It’s imperative to put patients and consumers before Big Pharma profits.’

The ‘Health Equity for Down Syndrome,’ ‘Same Access for Down Syndrome,’ and ‘Discriminate Against Down Syndrome’ posts apparently originate with a coalition of patient advocacy organizations. On February 4, 2022, the National Down Syndrome Society (NDSS) posted the following comment on its website:

*Down Syndrome Affiliates in Action (DSAIA), Down Syndrome Medical Interest Group (DSMIG-USA), GiGi’s Playhouse Down Syndrome Achievement Centers, Global Down Syndrome Foundation (Global), LuMind IDSC Foundation (LuMind IDSC), National Down Syndrome Congress (NDSC), National Down Syndrome Society (NDSS), and National Task Group on Intellectual Disabilities and Dementia Practices (NTG) collaborated to catalyze a community-wide response to the draft decision. Because of how significant the impact of Alzheimer’s disease is on our community, it’s critical that we come together and share a common message to combat this discrimination and protect our community’s access to treatment for Alzheimer’s disease.*  ^100^

While the organized effort is evident, and commentors submitting duplicates did name many of these organizations in their comments, we were not able to find the original sources of the duplicate text. It is, however, possible to find the parent text online, which suggests advocacy may have occurred via channels that are not publicly accessible or that websites have changed over time. For instance, At Her Own Pace, a parent-run blog dedicated to promoting Down Syndrome awareness and offering advice to families,^101^ has a blog post dated February 3, 2022 that discusses the proposed NCD. The poster began by noting that her daughter ‘has a 90% chance of developing Alzheimer’s disease (AD) simply because she has three copies of her 21^st^ chromosome.’^102^ She then explained that ‘[t]he drafted CED for aducanumab as it stands now excludes people with Down syndrome and other intellectual and developmental disabilities (I/DD), which means down the line having not been included in these trials [and perhaps] further exclusion from coverage.’^103^ Readers are then encouraged to submit comments to CMS. Three sample comments—‘Health Equity for Down Syndrome,’ ‘Same Access for Down Syndrome,’ and ‘Discriminates Against Down Syndrome’—are included in the post and described as ‘NDSS-provided.’^104^

## V. DISCUSSION

We analyzed nearly 10,000 comments submitted to CMS on the proposed NCD for mAbs directed against amyloid for treatment of AD.^105^ The vast majority, nearly 80%, of commentors expressing any opinion on CMS’s proposed use of CED, objected to *any* Medicare coverage of drugs in this class. Support for full coverage and support for CED were minority viewpoints. Notably, commentors who included segments of text from a form letter in their submission were much more likely to oppose any Medicare coverage than were commentors who submitted wholly unique comments; also found differences in commentors’ stated position on CED based on stakeholder type. The aducanumab controversy sat at the intersection of multiple contentious issues—including FDA drug approval standards, drug pricing, CMS coverage, AD as a public health issue, and health equity—and this was reflected in the comments.

CMS is legally required to review all comments and respond to them in its final decision, which must be issued no later than 60 days after the end of the public comment period.^106^ Given the substantial effort required to review public comments, the relevant agency is often the only entity to do so.^107^ Yet, an over-the-shoulder look at the agency’s review, even if after the fact, has value. Most importantly, it serves as a check on the government, confirming or refuting the accuracy of the agency’s overall interpretation. This check may be particularly important when, as with mAbs directed against amyloid for treatment of AD, the issue is hotly contested and the stakes are substantial. Our findings are, at a high level, similar to those reported by CMS and summarized in Table 1. CMS did not share its methodology, which may explain discrepancies between our findings where they exist.^108^

Importantly, our study does not merely validate CMS’s summary of the public comments; it also provides a more granular review of those comments and nuanced insights. We had the benefit of far more than 60 days to conduct our analyses. Our approach was both inductive, in that we based our analysis on our descriptive observation of the data, and deductive, in that we brought frameworks and ideas seen in the academic and public discussion to bear on the data. We were interested in identifying who submitted comments and in considering at greater length what the comments revealed about the proposed NCD as well as larger debates. Our analysis underscores the ways in which people viewed the public comment period as an opportunity to discuss not just the proposed NCD but numerous related issues, many of which are not within the purview of CMS and so fell outside the agency’s analysis.

In this section, we first look at differences within and across stakeholder groups, including their perspectives on key issues, as well as whether and why they submitted unique or duplicate comments; this is a novel contribution, as CMS did not report on stakeholder type. We then discuss how the themes we identified intersected with broader law and policy debates, including those informed by subsequent developments since the NCD was finalized.

### V.A. Stakeholder Perspectives

CMS did not, in its decision memo, distinguish between stakeholder groups but rather spoke of stakeholders as a singular group. As summarized in Table 2, we captured which stakeholder groups were represented within the public comments, as well as the frequency with which members of those groups submitted comments. Further, we looked for associations between stakeholder groups, the kinds of comments they submitted, and their viewpoints.

#### 1. Stakeholder Group Viewpoints

Our analysis offers evidence in support of what was suggested by the broader public discourse surrounding mAbs directed against amyloid for treatment of AD: stakeholder groups have distinct interests and so perceived themselves to be affected by the proposed NCD in distinct ways. These differences were evident in the issues stakeholder groups chose to highlight when commenting. For example, pharmaceutical companies discussed the potential downstream effects of CED on innovation more frequently than other stakeholders. Similarly, individuals who identified as Medicare beneficiaries were far more likely than others to discuss the Part B premium increases that were adopted to address the anticipated costs of covering aducanumab.

Looking at the comments submitted by members of a single stakeholder group, there was generally agreement regarding the group’s interests or goals, though no group was monolithic in its views. One issue proved especially divisive: family members and caregivers for persons with AD were almost evenly divided over whether aducanumab offered hope (a positive) or false hope (a negative). Looking to history, this heterogeneity is unsurprising. The accelerated approval pathway was created in 1992 as a response to the HIV/AIDS crisis; yet, the pathway’s emergence divided the HIV/AIDS advocacy community into those who sought to prioritize access and those who sought to ensure adequate evidence of effectiveness and safety.^109^ FDA officials justified aducanumab’s approval, in part, by appealing to patient voices that favored accepting uncertainty to secure access, given the lack of alternatives in the face of a terrible disease.^110^ Yet, we see in the public comments that patients and families do not uniformly see the tradeoff of certainty for speed as wise. Regulators and payers therefore should not assume uniformity^111^ or that a small group of patients can represent the full range of views held by an entire patient population. Policymakers must also consider that the patients and family members able to submit comments and make their voices heard today likely have interests distinct from *future* patients who could be expected to put more weight on certainty over speed^112^ but who cannot advocate on their own behalf because they don't yet exist.

Looking across stakeholder groups, we find areas of relative consensus as well as tensions. Consensus was, for example, evident in the widespread agreement that AD is a terrible disease and also that there are well-documented racial and ethnic disparities in its burden. By contrast, Medicare beneficiaries’ interest in controlling costs was clearly at odds with pharmaceutical companies’ interest in protecting profits. In both cases, commentors had a clear financial interest in advocating for their desired outcome.

Together, our findings suggest a reason to consider commentors’ stakeholder group affiliation when reviewing public comments: stakeholders’ interest in the outcome of a debate or decision-making process may influence (consciously or not) how they weigh evidence and formulate arguments.^113^ CMS is sophisticated enough that it was surely aware of the myriad interests at stake and the roles stakeholders’ interests played in shaping comments, though it did not explicitly address them. The motivations behind comments posted by individuals who do not identify with any stakeholder group may be harder to parse. To facilitate appropriate consideration of stakeholder interests in the public comment process, it may be appropriate to require commentors to state a stakeholder group affiliation or affiliations.

#### 2. Missing Stakeholder Groups

One purpose of a public comment period is to allow members of the public an opportunity to participate and provide feedback on an administrative action that will affect them. Thus, describing who submitted comments provides valuable insights into who CMS was and was not hearing from as it finalized its decision. People living with AD had the potential to be profoundly affected by the NCD, given that aducanumab was FDA-approved for the treatment of MCI or mild dementia caused by AD. Yet people living with AD are conspicuously missing from the public comments. Only seven individuals explicitly identified themselves as part of this stakeholder group, though more than six million Americans currently live with AD.^114^

One possibility is that, because AD is highly stigmatized,^115^ people living with AD submitted comments without disclosing their Alzheimer's status. If so, they would be included among the majority of commentors who did not specify a stakeholder group affiliation. However, this is unlikely to explain the near total absence of people living with AD. Another possible explanation is that AD is a neurodegenerative disease that affects cognition, and cognitive impairment is a predictable barrier to commenting, which is a relatively demanding task when one considers the numerous steps between realizing one can submit a comment and finally clicking ‘submit.’ This does not fully explain the absence of commentors living with AD, however, given that those with early stage disease are substantially less impaired. Nevertheless, the lack of comments from people living with AD may raise concerns about inclusion and the accessibility of commenting.

People living with AD usually have a caregiver who speaks on their behalf. These caregivers are often deeply invested in treatment decisions, and research suggests many are willing to tolerate significant treatment-associated risks for a chance to slow the progression of disease.^116^ Thus, caregivers might reasonably have been expected to hold strong opinions about the proposed NCD and to wish to engage with CMS, whether for themselves or as surrogates. And indeed, family members and caregivers of people living with AD comprised the third largest group of self-identified stakeholders. This helps to mitigate concerns about the absence of comments from persons living with AD. However, the number of family member and caregiver comments (n = 500) seems low relative to the population of ‘more than 11 million Americans [who] provide unpaid care for a family member or friend with dementia.’^117^ Caregivers for people living with AD experience spillover stigma,^118^ and some may have chosen not to disclose their caregiver status for this reason. A more likely explanation is that a substantial proportion of caregivers for individuals with MCI and early-stage dementia—that is, individuals potentially eligible for aducanumab—report facing significant burden. Faced with daily caregiving responsibilities, caregivers may be unaware of the opportunity to submit public comments or, if aware, unable to find the time to do so.

The barriers facing these two stakeholder groups – people living with AD and their caregivers—raise a question: how should CMS interpret and utilize a body of public comments when it cannot assume that those comments meaningfully reflect the views of an important community or communities?^119^ In considering this question, it is essential to recognize that public comments are not purported to be representative of relevant stakeholder views, nor are they handled as a mere tally of ‘votes.’ Agencies seeking public comment appropriately may weigh comments differently depending on the type of stakeholder making them and their respective expertise and interests, even if those comments are low in number or not the majority. Moreover, agencies likely recognize that there are many barriers to participating in the public comment process that may lead to overrepresentation of some stakeholders’ views and underrepresentation of others. While policymakers may consider efforts to improve accessibility to and input from heavily impacted groups in certain circumstances, such as educational sessions, outreach, surveys, and the like, these approaches can be resource-intensive and may only have a modest impact. Therefore, the key obligations of policymakers are to ensure the opportunity to be heard and to focus on the strength of the information and arguments provided by the public, rather than giving undue weight to the volume of certain perspectives.

#### 3. How Stakeholder Groups Engaged

Looking across stakeholder groups, patterns become evident in the content and style of presentation, as well as in which stakeholder groups were mobilized to submit public comments.

##### i. Sophistication of Comments and Epistemic Justice

CMS states in its decision memo that it finds ‘[p]ublic comments that give information on unpublished evidence such as the results of individual practitioners or patients … less rigorous and therefore less useful for making a coverage determination.’^120^ This statement is consistent with prior research suggesting that agency responsiveness is proportionate to the sophistication of the information—that is, the reasoning, data, and analysis—contained in comments.^121^ It is also appropriate for policymaking in areas that should be informed by scientific evidence to be guided by the highest quality science. Yet, scientific evidence alone cannot make challenging policy judgments, which call for weighing additional normative considerations, such as patients’ and caregivers’ tolerance for risk, uncertainty, opportunity costs, and public preferences about how resources are spent. Public comments are important because they are likely to help supply and draw attention to the additional non-scientific considerations that should factor into policy judgments. It is therefore possible to grant that some issues demand assessment by scientific, clinical, and statistical experts, given their unique qualifications, without discounting the perspectives of patients and caregivers.

In the context of this CMS decision, patients, family members, and caregivers have intimate knowledge of the problems associated with AD and their lived experience offers unique knowledge and insight^122^ about topics such as: the real world need for effective AD treatments; the desire to access promising interventions; the willingness (or not) to accept uncertainty about benefits and risks; the barriers to clinical trial participation; and the impact of out-of-pocket payments. These insights are partly why patient and family engagement is increasingly viewed as a broad ethical imperative, alongside the importance of giving those most affected by a decision a seat at the table where decisions are being made.

Although patient and family engagement is essential, other perspectives and types of expertise are also important.^123^ While patients can help explain why a particular clinical trial result would make a meaningful difference in their lives or why a particular set of risks are outweighed by benefits, their insights cannot change negative trial outcomes and their desperation for treatment cannot render an ineffective drug effective. Similarly, patients do not necessarily have special insights into whether CMS’s statutory standard for coverage has been met. They may even be described as having a conflict of interest—that is, a strong desire for coverage even if it means fewer resources available for others.

Thus, there is no easy answer as to how CMS ought to weigh patients’ and caregivers’ lived experience against the other types of experience and expertise. Providing commentors with more guidance, and perhaps asking specific questions for feedback from patients and caregivers based on their unique experiences, would help them shape their input in a more impactful way. More broadly, being clear and specific about what public input will be most helpful to policy decisions may help avoid patient and public engagement being merely performative on the part of agencies inviting public engagement and minimize frustration when policymakers advance approaches that are contrary to patient advocacy.

##### ii. Duplicate Comments – Down Syndrome

CMS reported, and we similarly found, a substantial number of duplicate comments. As detailed in § IV.B*,* duplicates resulted from organizations mobilizing their members and supporters. CMS did not explicitly distinguish between unique and duplicate comments in its decision memo.

Since the advent of electronic rulemaking, some people have applauded the potential for enhancing public participation, while others have worried that duplicate comments can spam agencies, turning rulemaking into an unmanageable process.^124^ Looking at comments submitted to the Environmental Protection Agency, Balla and colleagues found, contrary to both accounts, that ‘for better or for worse – neither rulemaking procedures nor outcomes are as a general matter responsive to information communicated through [mass comment campaigns].’^125^

In this data set, we see what looks to be an exception. More than 1000 comments—over 10% of the total submitted to CMS—came from self-identified family members and caregivers of persons with Down Syndrome. This number is notable because, based on birth prevalence, the population of people with Down Syndrome in the United States is estimated at 400,000.^126^ In this case, the comment campaign, which brought in supportive comments from others in addition to family members and caregivers, successfully influenced CMS. The agency explicitly noted that it removed the proposed exclusion of people with Down Syndrome and other I/DD ‘based on public comments.’^127^ How might this success be explained? Though it is generally more important for agencies to consider relevant information than stakeholder values, this NCD process was replete with value-laden issues such as whether justice required equal access for everyone. These were precisely the kind of issues raised in the Down Syndrome duplicates. Further, there was near unanimous agreement (among those who spoke to it) that barriers to access for people with Down Syndrome were discriminatory and unjust.^128^

##### iii. The ‘Missing’ Duplicates – Alzheimer’s Disease

Successful mobilization of supporters by Down Syndrome organizations makes the relative dearth of comments from AD patients, family members, and caregivers, discussed above, more noteworthy. The Alzheimer’s Association, a sophisticated and influential patient advocacy organization, surely could have organized a mass comment campaign had it desired to do so, especially given that it had ‘enthusiastically’ welcomed aducanumab’s ‘historic FDA approval.’^129^ In its 2023 annual report, the Association describes itself as ‘the leader in the fight to end [AD’s] devastation’ before stating that ‘[t]ogether with our hundreds of thousands of advocates, we are relentless in our efforts to make Alzheimer’s a national priority while speaking up for the needs and rights of people living with the disease.’^130^ In 2023, the Association reported more than $432 million in revenue.^131^

Indeed, prior to CMS’s public comment period, the Alzheimer’s Association had not shied away from being a prominent voice in favor of aducanumab. For instance, in October 2020, the Association submitted a letter to FDA supporting the drug’s approval, while also failing to disclose in the letter that the Association had received at least $1.4 million from Biogen and its partner, Eisai, since fiscal year 2018.^132^ In the face of criticism, the Association denied that it was unduly influenced by this financial support, though concern remained.^133^ Following the November 2020 meeting of FDA’s Peripheral and Central Nervous System Advisory Committee, the Alzheimer’s Association organized a closed-door discussion between senior FDA officials and patients, families, and Association representatives, based on concerns that ‘the voices of those diagnosed with Alzheimer’s and their caregivers had not truly been heard’ during the Advisory Committee meeting.^134^ Discussing aducanumab after that meeting, Office of New Drugs Director Peter Stein acknowledged that the FDA ‘heard very clearly from patients that they’re willing to accept some uncertainty to have access to a drug that could provide meaningful benefit.’^135^

FDA meetings with patient advocacy groups receiving industry funding raise concerns that pharmaceutical companies are using this approach as another channel for promoting their regulatory agendas.^136^ Relatedly, when advocacy organizations accept funding from companies, they may face decisions that pit funders’ interests against the interests of the organization’s constituents, creating conflicts of interest.^137^ For these reasons, the advocacy of the Alzheimer’s Association and other organizations around aducanumab highlighted the ways in which ‘[t]he structure of advocacy groups and the haphazard manner in which they wield their immense power are problematic.’^138^

Following FDA approval, the Alzheimer’s Association initially applauded CMS’s announcement of an NCD,^139^ but then publicly opposed both the draft^140^ and final NCD.^141^ In a statement released on the day the public comment period for the proposed NCD opened, the Association characterized the proposal as ‘shocking discrimination against everyone with Alzheimer’s disease, especially those who are already disproportionately impacted by this fatal disease, including women, Blacks and Hispanics.’^142^ This statement includes ideas subsequently found in comments submitted to CMS. For example, the statement included the claim that ‘[p]eople living with Alzheimer’s disease deserve the same access to therapies given to those living with other conditions like cancer, heart disease and HIV/AIDS,’ which was echoed by commentors (§ IV.A.5).

In stark contrast to the approach the Association took to support FDA approval of aducanumab and despite then-CEO of the Alzheimer’s Association Harry Johns claim that the organization would ‘use all avenues of communication to ensure those affected, the broader public and the administration truly understand the ramifications of this … [CMS] draft decision … ,’^143^ we found no evidence that the Association sought to mobilize a community response to CMS beyond submitting its own public comment. As of January 14, 2022—three days into the 30-day public comment window—visitors to the Alzheimer’s Association website would have seen a link to learn more about ‘Medicare’s Discriminatory Coverage Decision.’ Those who clicked this link would have seen a short statement on the CMS draft decision and, at the very bottom of the page, been encouraged to ‘Send a Message to President Biden’ via a hyperlinked form for emails or Tweets. Nowhere did the website mention that visitors could submit comments to CMS directly.^144^

It is unclear why the Association took this approach. However, one hypothesis is that it concluded a public comment campaign would be less effective than going over CMS’s head to Congressional offices, which are especially responsive to public pressure from their constituents and large interest groups. In addition to their in-house expertise and connections, large advocacy organizations retain lobbyists and regulatory affairs consultants, creating substantial access to the people they seek to influence. This could help explain why the Down Syndrome patient advocacy groups were more active in these public comments to CMS, given that they have only a fraction of the resources and access of an organization like the Alzheimer’s Association. Stated otherwise, public comment campaigns may be a tool of the (relatively) weak.

### V.B. Beyond the Proposed NCD

Our analysis underscores the ways in which people saw the public comment period as a forum to discuss both the proposed NCD and broader, related issues. CMS acknowledged many of these broader comments without substantively engaging with them in its decision memo. While this was appropriate for CMS’s purposes, the comments offer insights regarding public perceptions of FDA’s approval standards, the relationship between regulators and payers, approaches to evidence generation, and health equity, all of which reflect important current health law and policy debates.

#### 1. FDA’s Approval of Aducanumab

One of the themes highlighted by CMS was ‘lack of evidence for clinical benefit’ of mAbs for AD. Elaborating, the agency wrote: ‘The majority of public comments stated that that there is not enough evidence showing that these drugs/biologicals provide a clinical benefit in order to cover antiamyloid mAbs.’^145^ We similarly found that a substantial number of comments addressed the (in)adequacy of evidence that aducanumab is safe and effective, and we agree with CMS’s assessment that the majority perspective was that evidence of clinical benefit was lacking.

Drug approval is outside CMS’s mandate, and accordingly, CMS focused on how this perceived lack of evidence informed commentors’ views on the agency’s proposed NCD. However, the comments also provide insight regarding public perception of the FDA’s decision to grant accelerated approval to aducanumab—insight that may otherwise not have found a public channel, given the lack of public processes at FDA following aducanumab’s approval.

FDA’s decision was unpopular in many quarters. Some individuals submitting unique comments and many submitting duplicate comments focused on accelerated approval, expressing concerns specific to aducanumab’s accelerated approval or about the pathway more generally. Commentors did not, however, address delays in the completion of confirmatory trials following accelerated approval^146^ or FDA’s poor record of withdrawing drugs that fail to confirm benefit after accelerated approval,^147^ both of which have been part of broader recent criticism of the accelerated approval pathway.^148^

Commentors also raised concerns about FDA’s motivations for approving aducanumab, focusing on the close collaboration between FDA and Biogen during the approval process and raising broader concerns about regulatory capture, which were the source of later Congressional investigation.^149^ While some commentors saw FDA’s approval as surprising for an agency that ‘was historically trusted to make good medical decisions,’ others characterized the decision as compounding a pattern of growing reputational harms to the agency. The idea of reputational harm was seen most frequently in the duplicate comment ‘Galling Disregard for Science,’ but was also present among unique comments.

Commentors’ concerns about accelerated approval and regulatory capture are echoed in some of the changes made to the accelerated approval pathway in the Food and Drug Omnibus Reform Act of 2022 (‘FDORA’). Indeed, the controversies surrounding aducanumab’s approval contributed to the political tipping point that allowed these reforms to be passed.^150^ FDORA authorizes FDA to require that confirmatory studies be underway prior to granting accelerated approval, an approach intended to minimize delay in completing these studies but one the agency did not require for aducanumab. The law also sought to reinforce the procedures for expedited withdrawal of approval if confirmatory studies fail to show a product’s clinical benefit.^151^ While these are important steps, FDORA’s reforms excluded more radical structural changes needed to restore commitment to scientific rigor at FDA.^152^ Thus, while aducanumab served as a crystallizing issue for commentors, many of the concerns they raised have broader, unresolved implications for FDA policy. Relatedly, the question of who should bear responsibility for post-approval evidence generation (and associated costs), an issue raised by many commentors, remains a live issue not addressed by FDORA reforms.^153^

#### 2. The Relationship Between CMS and FDA

Commentors did not always appreciate that CMS and FDA play distinct roles in patient access (§ IV.A.4). Some argued that the agencies must move in lockstep and that CMS was overstepping its bounds by limiting coverage after FDA approval, a belief likely driven by the fact that CMS has rarely employed CED for drugs.^154^ Other commentors appreciated that each agency has the authority to come to its own determinations, seeing CMS’s approach as corrective of FDA’s mistaken approval of aducanumab.

There is a clear legal difference between FDA approval, which is based on a standard of safety and effectiveness (without considering comparative effectiveness or magnitude of clinical benefit), and CMS coverage, which is based on a standard that considers whether a drug is reasonable and necessary for the Medicare population. In April 2022, FDA Commissioner Robert Califf and CMS Administrator Chiquita Brooks-LaSure issued a joint statement indicating that ‘[o]ur agencies remain committed to using our distinct set of authorities to ensure the continued availability of medical products that meet our respective standards to care for the people we serve.’^155^ The FDA commissioner reiterated this stance in June 2023, when he again defended CMS’s independent role in determining drug coverage following FDA approval.^156^

This approach has been upheld in court, with judicial decisions showing ‘deference in upholding Medicare’s authority to limit coverage of FDA-approved products.’^157^ It is unclear, however, whether this trend will continue following the Supreme Court’s 2024 decision in *Loper Bright Enterprises v. Raimondo* to reject the previously foundational principle of *Chevron* deference, under which courts were directed to defer to reasonable agency interpretations of ambiguous statutes.^158^ Under *Loper Bright*, federal courts may not be bound to CMS’s interpretation of ‘reasonable and necessary,’ raising the possibility of future judicial findings that FDA-approved drugs are, by definition, ‘reasonable and necessary’ or that CMS may not use its NCD authority to make determinations about classes of drugs rather than specific products.^159^  *Loper Bright* is part of a broader judicial trend toward reducing agency authority, which will likely lead to more litigation challenging agency decisions,^160^ forcing substantial expenditure of agency resources, and likely inhibiting thoughtful agency responses. Relatedly, the public comment process may contribute to this concern, creating opportunities for motivated parties to challenge agency approaches in preparation for litigation.^161^ The newly unsettled administrative law landscape also provides judges with opportunities to find fault with agency procedures (ie failure to adequately engage with all comments) to justify overturning policy decisions.

One solution would be for Congress to explicitly grant CMS statutory authority for its CED program, which would validate CMS’ approach while protecting its discretion to tailor coverage determinations even following FDA approval.^162^ Yet, this seems unlikely. Some in Congress excoriated CMS’s CED policy for mAbs directed against amyloid for treatment of AD and proposed legislation to reign the agency in.^163^ There is ongoing debate about whether FDA approval should be the determining factor for payer coverage or whether the U.S. payment model should move toward the Health Technology Assessment (HTA) approach, which summarizes information about medical, economic, social, and ethical issues related to health technologies and is used in peer countries.^164^

#### 3. Downstream Effects of the Proposed NCD on Drug Development and Evidence Generation

In its decision memo, CMS noted that ‘[s]ome commenters stated that the CED requirement would hurt future innovation for [AD] treatments,’ but indicated that it did not agree with those comments.^165^ CMS highlighted that the value of its approach was to ‘[give] the public a clear definition of where the goal line is for what constitutes success in a trial’ and claimed that simply ‘covering a drug that has not been shown to be effective [without requiring evidence development] may incentivize production, marketing and sales of similarly ineffective drugs, at the cost of hard research to find ones that do provide a clinical benefit to patients.’^166^

As we highlighted in § IV.A.4, nearly all the comments regarding potential effects of the proposed NCD on future research expressed a belief CED would negatively affect drug development, a claim also found in some academic literature.^167^ These comments warned that pharmaceutical companies would not ‘continue to invest money in developing a treatments [sic] that will have no future in reaching the market.’ These comments were not limited to those submitted by pharmaceutical companies and industry associations, who might (in CMS’s view) be incentivized to invest in ‘ineffective drugs,’ but were shared by clinicians, academics, and other stakeholders. Although it is too soon to assess the true impact of this NCD on innovation, it is worth noting that the FDA has granted traditional approval to two mAbs directed against amyloid for treatment of AD, donanemab and lecanemab, since the NCD was finalized. Consistent with the NCD, Medicare covers these drugs when ‘a prescribing clinician or their staff decides the Medicare coverage criteria are met and submits information to help answer treatment questions in a qualifying study,’ which has taken the form of a registry.^168^ Donanemab and lecanemab were well along the development trajectory when the NCD was finalized, such that it likely had little impact on corporate investment decisions regarding those drugs, both of which face important ongoing questions regarding the magnitude of their benefits and risks.^169^ Going forward, the impact of the CED approach on innovation should be carefully evaluated, considering the volume, type, and quality of drugs brought to market.

In its decision memo, CMS did not explicitly engage with commentors concerned that the effects of the proposed NCD would be felt beyond AD, including in rare disease research, where industry incentives for drug development are perceived as especially precarious. Such concerns tied into worries that CMS’s decision would undermine FDA’s accelerated approval pathway by requiring additional trials for evidence development prior to full Medicare reimbursement. In this way, the goal of accelerated approval to provide early patient access despite residual uncertainty about drug benefit might be undercut, since patient access usually depends on payer coverage. Yet, as discussed below, there are contrary arguments that pricing and payment should be adjusted in alignment with the level of evidence to support a drug and the magnitude of its benefit, an approach that could help achieve accelerated approval’s goals by incentivizing rapid confirmation of effectiveness.^170^

Select commentors identified cancer and HIV as two conditions for which accelerated approval has played an especially significant role in expediting access to promising therapies. The reality is more nuanced. It is true that accelerated approval had ‘its origins in HIV treatment … but is now most common in oncology, with approximately one-third of all oncology drug approvals using the pathway and more than 80% of all accelerated approvals being granted for cancer therapies.’^171^ Yet, studies of accelerated approvals in oncology have raised questions about how often these drugs truly help patients live better or longer, even when they are converted to regular approval, due to delays in completing confirmatory studies, weak study designs and endpoints used in those studies, and allowing drugs to remain on the market even when benefit is not confirmed.^172^ This offers important context for commentors’ assertions that CMS was discriminating against AD patients by treating them differently than patients in these other disease areas. Equal treatment does not necessarily mean less CED. Perhaps CMS should be using CED more frequently, for example in oncology, rather than less.

In this vein, commentors also voiced concern that new drugs in cancer and other disease areas would be subject to similar scrutiny by CMS going forward. We note that many of these concerns echo those raised by industry and some rare disease advocacy groups about Medicare drug price negotiation under the Inflation Reduction Act of 2022 (‘IRA’).^173^

#### 4. Downstream Effects of the Proposed NCD on Health Equity

There was substantive agreement between CMS’s summary of concerns about the repercussions of CED on health equity and the issues identified in our review (§ IV.A.4). There is good reason to highlight equity concerns in this context, as Black and Hispanic older adults are significantly more likely to develop AD than white older adults but are also more likely to be excluded from clinical trials. The lack of representation in clinical trials—and the need to include minority populations—is well recognized within the AD research community.^174^

CMS took several steps to address commentors’ equity-related concerns. As noted above, CMS made changes between the proposed and final NCD so as not to exclude beneficiaries with Down Syndrome from participating in CED studies. Additionally, CMS updated the study requirements, which had initially required only that ‘[t]he diversity of patients included in each trial … be representative of the national population diagnosed with AD,’^175^ to the more rigorous requirement that study protocols and analysis plans must include a study population ‘whose diversity of patients are representative of the national population with MCI due to AD or mild AD dementia.’^176^ CMS also removed its proposed requirement that all trials be conducted in hospital-based outpatient centers, responding to commentors’ worries that this requirement would harm members of minority racial and ethnic groups as well as people residing in rural areas.

Some commentors expressed concerns that CED would contribute to inequity by creating different access to treatment depending on one’s ability to pay. In brief, their argument was that the proposed NCD ‘would allow Medicare to make patients chose between enrolling in a clinical trial, paying out of pocket for the drug, or foregoing the drug altogether.’^177^ Biogen and major Alzheimer’s patient advocacy groups—many of which received money from drug makers—similarly argued that the proposed NCD would make it harder for vulnerable populations to access aducanumab and set a poor precedent for future drugs.^178^

Invocations of health disparities to argue in favor of aducanumab coverage were largely rejected by Alzheimer’s researchers and others, who variously described them as ‘like a betrayal’ and ‘performative.’^179^ Such criticism stems from a perception that advocacy organizations perversely invoked equity to protect the interests of pharmaceutical companies like Biogen rather than to promote patient wellbeing, as well as a concern that equity arguments were being offered to support payer coverage even as only a small fraction of participants in the trials supporting aducanumab’s approval were drawn from minority groups.^180^ If equity was so important, why was it only being emphasized after approval? Overall, arguments rooted in health equity can be powerful, but also can be misused in the context of expensive, unproven interventions. While equitable access to beneficial drugs is an important health policy goal, the argument for equity falls flat when the product in question has not sufficiently demonstrated benefit, as was the case for aducanumab.^181^

#### 5. Aducanumab’s Cost, Value, and Affordability

CMS acknowledged that ‘[m]any commenters expressed their concern with the price of Aduhelm and frustration with the increase in Medicare premiums, stating that Medicare should negotiate the price for these drugs’ but did not engage with these comments in depth.^182^ The entirety of the agency’s response read: ‘As a policy matter, CMS does not consider cost when making an NCD. The Part B premium and drug pricing is outside the scope of this NCD.’^183^ CMS’s response is factually correct and avoiding these debates was likely a wise strategy as CMS made an already politically challenging decision. Nonetheless, recognition of the emotionally charged nature of many comments regarding cost and pharmaceutical company greed provides helpful insight into the nature of these challenges.

Some family members and caregivers of people living with AD noted the huge financial burdens associated with Alzheimer’s care, sometimes sharing painful anecdotes from their own experience—the type of unique lived experience that can be usefully shared in public comments. For them, the NCD process sharpened their disappointment that the agency can cover an expensive drug without clear evidence of benefit, like aducanumab, whereas the agency legally cannot pay for evidence-based services and supports that would be beneficial for beneficiaries with AD and their caregivers.^184^ Moreover, while aducanumab was indicated for a relatively narrow number of Medicare beneficiaries with AD, many more would benefit from, for example, insurance reimbursement for the services of a specialized dementia care manager.^185^

It is an open question whether Biogen could have averted much of the aducanumab controversy by simply pricing the drug lower from the start.^186^ The initial price, combined with the number of eligible patients, heightened scrutiny and exacerbated concerns about whether it should have been approved at all. Pricing is not intended to be a consideration in FDA review, and prior to the IRA, CMS was not permitted to negotiate prices for drugs covered by Medicare. This largely leaves companies to set their own prices, not based on the quantity or quality of evidence supporting the drug, nor on the level of clinical benefit provided to patients, but rather at whatever price the market will bear. Biogen chose an initial price of ~$56,000 per year, which it subsequently halved in response to slow uptake and decisions by some private payers not to cover aducanumab at the higher price.^187^

Although they did not make their way into FDORA as passed, reforms have been suggested that would limit payments for accelerated approval drugs until they transition to traditional approval based on stronger evidence of benefit.^188^ For example, in 2023, CMS put out a proposal for a pilot program under which it would adjust coverage of accelerated approval drugs to ‘reduce Medicare spending on drugs that have no confirmed clinical benefit,’ encourage timely confirmatory trial completion, and improve access to post-market safety and efficacy data.^189^ However, that pilot never launched and appears to be on hold or abandoned.^190^

It is important to note that the NCD public comment period predates the passage of the IRA, which authorizes the U.S. Department of Health and Human Services (‘HHS’) to negotiate prices with participating manufacturers for a select number of high-expenditure, qualifying single-source Medicare drugs.^191^ The fact that Medicare could not negotiate drug prices was a common point of contention for commentors, who called the lack of negotiation authority ‘crazy’ and urged Congress to ‘find a conscience’ and allow CMS to negotiate prices. While the IRA has been celebrated as a notable step toward lowering drug prices, its provisions are limited and do not immediately impact mAbs directed against amyloid for treatment of AD. Price negotiations began with only a small number of Part D drugs, and no drug is eligible for negotiation until at least 9 years after FDA approval.

One topic that was minimally addressed in the public comments was that state Medicaid programs are required to cover nearly all FDA-approved drugs, even those Medicare declines to cover (§ IV.A.6.i). Acknowledging that state Medicaid programs have ‘expressed concern regarding the budgetary effects of coverage requirements for prescription drugs – especially those that, like aducanumab, have been granted accelerated approval,’^192^ some have argued that Congress should take steps to protect state budgets. In 2023, the Medicaid and CHIP Payment and Access Commission, a non-partisan legislative branch agency,^193^ referenced aducanumab when calling for Congress to allow states to exclude or otherwise restrict coverage of a drug based on CED requirements implemented under a Medicare NCD.^194^ No such change has yet been enacted.

#### 6. Hope and False Hope

In its summary of comments, CMS noted that ‘[s]everal commenters stated that CMS should not restrict access to an FDA approved treatment for AD, especially since there are no alternative options available.’^195^ Replying, CMS acknowledged the ‘significant burden’ of AD while also stating that ‘[m]ore trials are needed, and the results of these trials will assist in providing answers to CMS, as well as to clinicians, patients, and caregivers, regarding the clinical benefits and harms of this treatment.’ Notably, CMS did not speak of ‘hope’ or ‘false hope,’ two terms that we saw frequently (§IV.A.7). Nor did CMS speak of autonomy, either for patients or for the clinicians treating them.

People living with AD have a life-limiting disease and limited treatment options. Understandably, some are willing to trade uncertainty about clinical benefit for access to a potentially effective drug. Hope is associated with important outcomes for health and well-being.^196^ But neither a desire for hope nor desperation are reasons for the FDA to approve a drug or for CMS to pay for one.^197^ Accepting patients’ preferences as determinative would dangerously erode the agencies’ respective regulatory and stewardship roles. A ‘something is better than nothing’ mindset is understandable for individuals, and we saw evidence of that mindset in comments such as ‘the potential for the medication to stop the ravages of Alzheimer’s disease far outweighs any possibility of side effects.’ Such a mindset is not, however, appropriate for gatekeepers like the FDA and CMS.

A substantial concern is that the FDA has increasingly adopted the ‘something is better than nothing’ approach through exercises of regulatory flexibility, accepting less and weaker evidence to support drug approval.^198^ This creates the deleterious illusion of regulation while disconnecting the agency from its public health mission,^199^ namely to ensure that drugmakers produce the evidence patients and clinicians need to meaningfully inform treatment decisions.^200^ Arguably, the FDA showed too much deference to patient desperation in approving aducanumab.^201^ As a result, it abdicated its responsibility as a gatekeeper to require evidence that drugs are safe and effective, ultimately shifting the responsibility for gatekeeping onto CMS. As the FDA’s regulatory flexibility intensifies, for example in the context of gene therapy and other domains,^202^ CMS will face growing pressure to cover drugs with a limited evidence base, an important policy challenge.

### V.C. Limitations

As is typical for qualitative methods, our codes and coding process may have been unique to our research team, with somewhat different results if performed by another group. Our method tended to be over- rather than under-inclusive. For instance, when stakeholders could be identified as belonging to two or more categories, we coded them to each category. Throughout, our data may differ from CMS data because CMS did not provide its methodology, precluding exact replication. More broadly, as noted above, public comments are not necessarily representative of the public’s views, but rather only of those who were aware of the opportunity and chose to comment.

## VI. CONCLUSION

Aducanumab has widely become recognized as one of the FDA’s greatest mistakes, a paradigmatic example that highlighted enduring and emerging challenges in drug approval and coverage. Thus, although Biogen pulled aducanumab from the market in 2024,^203^ the drug’s ongoing and outsized role in policy discussions makes it worthy of continued consideration.

This analysis of the nearly 10,000 public comments submitted to CMS in response to the agency’s proposed NCD adds significant depth to our understanding the stakeholders involved in the decision, their views, and how these views intersected with broader debates, as well as the pros and cons of public comment processes to inform policy judgments. Going forward, the FDA, CMS, and Congress should work together to address the many unresolved challenges highlighted by commentors, including the relationship between FDA approval and CMS coverage, the impact of drug approval and coverage on innovation and access, and how best to ensure that patients with terrible diseases have treatment options supported by strong evidence rather than hope alone.

## Footnotes

1 Philip Scheltens et al., *Seminar: Alzheimer’s Disease 2020*, 397 The  Lancet 1577 (2021).

2 Alzheimer’s Association Report: 2023 Alzheimer’s Disease Facts and Figures, Alzheimer’s & Dementia at 20 (2023), https://onlinelibrary.wiley.com/doi/10.1002/alz.13016 (last visited Mar. 19, 2023). In 2022, more than 11 million caregivers provided an estimated 18 billion total hours of unpaid care to people with Alzheimer’s or other dementias. *Id.* at 41.

3
*See, e.g.*, Emily A. Largent et al., *FDA Drug Approval and the Ethics of Desperation*, 181 Jama  Internal  Medicine 1555 (2021).

4 FDA Grants Accelerated Approval for Alzheimer’s Drug, fda.gov, June 7, 2021, https://www.fda.gov/news-events/press-announcements/fda-grants-accelerated-approval-alzheimers-drug; *see also* National Alzheimer’s Project Act, Pub. L. No. 111–375, § 2, 124 Stat. 4100, 4100 (codified at 42 U.S.C. § 11,225) (listing, as one of the purposes of the Act, ‘accelerat[ing] the development of treatments that would prevent, halt, or reverse the course of Alzheimer’s … .’).

5 Jennifer Rose V. Molano, *The Aducanumab Controversy – How Do Clinicians Proceed?*, Nejm  Journal  Watch (2021), https://www.jwatch.org/na54036/2021/09/29/aducanumab-controversy-how-do-clinicians-proceed.

6
*See, e.g.*, Jason Karlawish, *If the FDA Approves Biogen’s Alzheimer’s Treatment, I Won’t Prescribe It*, stat (May 30, 2021), https://www.statnews.com/2021/05/30/if-the-fda-approves-biogens-alzheimers-treatment-i-wont-prescribe-it/.

7 Aaron S. Kesselheim & Jerry Avorn, *Letter from Aaron S. Kesselheim, Professor of Med., Brigham and Women’s Hosp./Harvard Med. Sch., to Janet Woodcock, Acting Comm. of Food and Drugs* (2021), https://pbs.twimg.com/media/E3jKN4GWYAUGj9U.png.

8 42 U.S.C. § 1395y.

9 Medicare Program; Revised Process for Making National Coverage Determinations, 78 Fed. Reg. 48164, 48165 (2013).

10 Medicare Coverage Determination Process, cms.gov, https://www.cms.gov/medicare/coverage/determination-process (last visited Apr 8, 2023).

11 Medicare Administrative Contractors, cms.gov, https://www.cms.gov/medicare/coding-billing/medicare-administrative-contractors-macs (last visited Jun 3, 2024).

12 Local Coverage Determinations | CMS, https://www.cms.gov/medicare/coverage/determination-process/local (last visited Jun 3, 2024).

13 American College of Radiology, *National and Local Coverage Determinations*, American  College of  Radiology, https://www.acr.org/Advocacy-and-Economics/Radiology-Economics/Medicare-Medicaid/Coverage.

14 CMS Opens National Coverage Determination Analysis on Treatment for Alzheimer’s Disease, cms.gov, Jul. 12, 2021, https://www.cms.gov/newsroom/press-releases/cms-opens-national-coverage-determination-analysis-treatment-alzheimers-disease.

15
*See, e.g.*, American Academy of Neurology, *CMS Proposes Limited Coverage of Aducanumab – Patients Must Participate in Trials to Receive Treatment*, https://www.aan.com/siteassets/home-page/tools-and-resources/practicing-neurologist--administrators/aducanumab/summary-of-proposed-ncd.pdf (last visited Jun 3, 2024).

16 Monoclonal Antibodies Directed Against Amyloid for the Treatment of Alzheimer’s Disease (CAG-00460 N) - Proposed Decision Memo, cms.gov, https://www.cms.gov/medicare-coverage-database/view/ncacal-decision-memo.aspx?proposed=Y&NCAId=305 (last visited Mar. 18, 2024).

17 Medicare Coverage Document - Guidance for the Public, Industry, and CMS Staff: Coverage with Evidence Development, cms.gov, Nov. 20, 2014, https://www.cms.gov/medicare-coverage-database/view/medicare-coverage-document.aspx?MCDId=27 (last visited Mar 19, 2023).

18
*Id.*

19 At the time of Medicare’s proposed NCD, aducanumab was the only mAb directed against amyloid for the treatment of AD that had been approved by the FDA. As described below, 2 other mAbs for AD have since been granted traditional approval, lecanemab and donanemab.

20 Kathryn A. Phillips, *CMS Coverage With Evidence Development—Challenges and Opportunities for Improvement*, 3 Jama  Health  Forum e223061 (2022).

21 Emily Zeitler et al., *Coverage With Evidence Development: Where Are We Now?*, 28 Am. J. Managed  Care 382 (2022); Phillips, *supra* note 20. For a recent review of the largely deferential case law addressing CMS’s authority to implement CED, *see* C. Joseph Ross Daval & Aaron S. Kesselheim, *Authority of Medicare to Limit Coverage of FDA-Approved Products: Legal and Policy Considerations*, 183 Jama  Internal  Medicine 999 (2023).

22
*Cf.* Carlo Federici et al., *Coverage with Evidence Development Schemes for Medical Devices in Europe: Characteristics and Challenges*, 22 The  European  Journal of  Health  Economics 1253 (2021).

23 Monoclonal Antibodies Directed Against Amyloid for the Treatment of Alzheimer’s Disease (CAG-00460 N) - Decision Memo, cms.gov, https://www.cms.gov/medicare-coverage-database/view/ncacal-decision-memo.aspx?proposed=N&NCAId=305 (last visited Mar 18, 2024).

24 Prior to this public comment period on the specific proposal, CMS had solicited comments in an initial comment period in July and August of 2021 and held meetings with patient advocacy organizations, manufacturers, payers, and other stakeholders. *See* Monoclonal Antibodies Directed Against Amyloid for the Treatment of Alzheimer’s Disease (CAG-00460 N) - Decision Memo, CMS.gov, https://www.cms.gov/medicare-coverage-database/view/ncacal-decision-memo.aspx?proposed=N&NCAId=305 (last visited Mar. 18, 2024).

25
*Id.*

26 5 U.S.C. § 706(2)(A).

27 Matthew S. McCoy et al., *Patient and Public Involvement: Two Sides of the Same Coin or Different Coins Altogether?*, 33 Bioethics 708 (2019).

28 Nina A. Mendelson, *Democracy, Rulemaking, and Outpourings of Comments*, The  Regulatory  Review, Dec. 20, 2021, https://www.theregreview.org/2021/12/20/mendelson-democracy-rulemaking-and-comments/.

29 Owen Dyer, *Aduhelm: Biogen Abandons Alzheimer’s Drug after Controversial Approval Left It Unfunded by Medicare*, 384 BMJ q281 (2024). Aduhelm brought in only $7.8 million in its first year and a half on the market. *See* Rebecca Robbins, *Biogen Abandons Its Controversial Alzheimer’s Drug Aduhelm*, The  New  York  Times, Jan. 31, 2024, https://www.nytimes.com/2024/01/31/business/biogen-alzheimers-aduhelm.html.

30 Biogen to Realign Resources for Alzheimer’s Disease Franchise, biogen.com (Jan. 31, 2024), https://investors.biogen.com/news-releases/news-release-details/biogen-realign-resources-alzheimers-disease-franchise.

31
*Id.*

32 FDA Converts Novel Alzheimer’s Disease Treatment to Traditional Approval, fda.gov (Jul. 6, 2023), https://www.fda.gov/news-events/press-announcements/fda-converts-novel-alzheimers-disease-treatment-traditional-approval.

33 FDA approves treatment for adults with Alzheimer’s disease, fda.gov (Jul. 2, 2024), https://www.fda.gov/drugs/news-events-human-drugs/fda-approves-treatment-adults-alzheimers-disease.

34 Reuters, *Alzheimer’s Drug Adoption in US Slowed by Doctors’ Skepticism*, US News & World  Report (Apr. 23, 2024), //www.usnews.com/news/top-news/articles/2024-04-23/alzheimers-drug-adoption-in-us-slowed-by-doctors-skepticism.

35
*Id.*

36 Robbins, *supra* note 29.

37
*See, e.g.*, Ali B. Abbasi et al., *Why Evidence Generation Should Matter to Payers and How They Can Help*, 332 Jama 412 (2024) (arguing that increasing private payer involvement in evidence generation can benefit medical innovation).

38 Monoclonal Antibodies Directed Against Amyloid for the Treatment of Alzheimer’s Disease (CAG-00460 N) - Decision Memo, CMS.gov, https://www.cms.gov/medicare-coverage-database/view/ncacal-decision-memo.aspx?proposed=N&NCAId=305 (last visited Mar. 18, 2024).

39 Public Citizen Statement: CMS Follows the Science, Makes the Right Call in Proposing Medicare Coverage for the Alzheimer’s Disease Drug Aducanumab Only for Beneficiaries Who Enroll in a Randomized Clinical Trial, Public  Citizen (2022), https://www.citizen.org/news/public-citizen-statement-cms-follows-the-science-makes-the-right-call-in-proposing-medicare-coverage-for-the-alzheimers-disease-drug-aducanumab-only-for-beneficiaries-who-enroll-in-a-random/; FDA approves treatment for adults with Alzheimer’s disease, FDA.gov (2024), https://www.fda.gov/drugs/news-events-human-drugs/fda-approves-treatment-adults-alzheimers-disease (last visited Nov 16, 2024).

40
*See, e.g.*, CMS Treatment Coverage Policy: What It Means for People Living with Alzheimer’s, Alzheimer’s  Association, https://www.alz.org/alzheimers-dementia/treatments/cms-medicare-coverage (last visited Apr. 9, 2023); Lauren Neves, *PhRMA Submits Comments Urging CMS to Withdraw Coverage Proposal*, The  Pharmaceutical  Research and  Manufacturers of  America (2022), https://phrma.org/Blog/phrma-submits-comments-urging-cms-to-withdraw-coverage-proposal.

41 Emily A. Largent et al., *Aspiring to Reasonableness in Accelerated Approval: Anticipating and Avoiding the Next Aducanumab*, 39 Drugs & Aging 389 (2022).

42 Biogen and Eisai to Discontinue Phase 3 ENGAGE and EMERGE Trials of aducanumab in Alzheimer’s Disease, biogen.com (2019), https://investors.biogen.com/news-releases/news-release-details/biogen-and-eisai-discontinue-phase-3-engage-and-emerge-trials (last visited Oct. 7, 2021).

43 These analyses would not be published in the peer-reviewed literature until March 2022. *See, e.g.*, Samantha Budd Haeberlein et al., *Two Randomized Phase 3 Studies of Aducanumab in Early Alzheimer’s Disease*, 9 J. Prev. Alzheimers  Dis. 197 (2022).

44 Biogen Plans Regulatory Filing for Aducanumab in Alzheimer’s Disease Based on New Analysis of Larger Dataset from Phase 3 Studies, biogen.com (2019), https://investors.biogen.com/news-releases/news-release-details/biogen-plans-regulatory-filing-aducanumab-alzheimers-disease (last visited Dec. 8, 2021); John Carroll, *In a Stunning Turnaround, Biogen Says That Aducanumab Does Work for Alzheimer’s—but Data Mining Incites Controversy and Questions*, Endpoints  News (2019), https://endpts.com/in-a-stunning-turnaround-biogen-says-that-aducanumab-does-work-for-alzheimers-and-theyre-prepping-a-pitch-to-the-fda/ (last visited July 21, 2021).

45 Pam Belluck, *F.D.A. Panel Declines to Endorse Controversial Alzheimer’s Drug*, The  New  York  Times, Nov. 6, 2020, https://www.nytimes.com/2020/11/06/health/aducanumab-alzheimers-drug-fda-panel.html (last visited Mar. 19, 2023).

46 Stephen Salloway et al., *Amyloid-Related Imaging Abnormalities in 2 Phase 3 Studies Evaluating Aducanumab in Patients With Early Alzheimer Disease*, 79 Jama  Neurology 13 (2021).

47 Kesselheim & Avorn, *supra* note 7.

48 G. Caleb Alexander et al., *Revisiting FDA Approval of Aducanumab*, 385 New  England  Journal of  Medicine 769 (2021).

49 Accelerated Approval, fda.gov, Feb. 24, 2023, https://www.fda.gov/patients/fast-track-breakthrough-therapy-accelerated-approval-priority-review/accelerated-approval (last visited Mar. 19, 2023).

50 Holly Fernandez Lynch et al., *Extending the US Food and Drug Administration’s Postmarket Authorities*, 4 Jama  Health  Forum e231313 (2023); *see also* Largent et al., *supra* note 41, at 389 (identifying normative judgments in key aspects of FDA’s implementation of accelerated approval and recommending procedural protections to promote reasonableness in this decisionmaking process).

51
Food & Drug  Admin., *Guidance for Industry: Expedited Programs for Serious Conditions - Drugs and Biologics*, (2014), https://www.fda.gov/media/86377/download (last visited Apr. 22, 2023); *but see* Joshua D. Wallach et al., *Comparison of Duration of Postapproval vs Pivotal Trials for Therapeutic Agents Granted US Food and Drug Administration Accelerated Approval, 2009–2018*, 4 Jama  Network  Open e2133601 (2021) (suggesting that the time in fact saved may be minimal).

52 Joshua J. Skydel et al., *Spending by the Centers for Medicare & Medicaid Services Before and After Confirmation of Benefit for Drugs Granted US Food and Drug Administration Accelerated Approval, 2012 to 2017*, 3 JAMA Health  Forum e221158 (2022). *See also* Christina Jewett, *F.D.A. Panel Recommends Pulling Preterm Birth Drug From the Market*, The  New  York  Times, Oct. 19, 2022, https://www.nytimes.com/2022/10/19/health/fda-preterm-birth-drug.html (discussing FDA’s decision to withdraw a preterm birth drug that failed to demonstrate efficacy after being granted accelerated approval and noting that the agency’s delay in withdrawing accelerated approval was ‘lucrative for the drugmaker’).

53 FDA Grants Accelerated Approval for Alzheimer’s Drug, *supra* note 4. Notably, FDA had never returned to the advisory committee to seek its input on accelerated approval of aducanumab. Although lecanemab also received accelerated approval on the basis of the same surrogate endpoint, and subsequently was granted traditional approval, the endpoint remains unvalidated.

54 Simon Makin, *The Amyloid Hypothesis on Trial*, 559 Nature S4 (2018).

55 Alexander et al., *supra* note 48, at 771.

56 Billy Dunn, Peter Stein & Patrizia Cavazzoni, *Approval of Aducanumab for Alzheimer Disease—The FDA’s Perspective*, 181 Jama  Internal  Medicine 1276, 1278 (2021). Following the advisory committee meeting, the Alzheimer’s Association, a patient advocacy organization, organized a discussion between senior FDA officials and patients, families, and Association representatives that may have influenced FDA’s decision. *See* Derrick Gingery, *Patient Support May Have Helped Push Aduhelm Toward Approval*, Pink  Sheet  Citeline  Regulatory (2021), http://pink.citeline.com/PS144438/Patient-Support-May-Have-Helped-Push-Aduhelm-Toward-Approval.

57 Jason Karlawish & Joshua D. Grill, *The Approval of Aduhelm Risks Eroding Public Trust in Alzheimer Research and the FDA*, 17 Nat. Rev. Neurol. 523 (2021).

58 Michael Carome, *Outrage of the Month: FDA’s Inappropriate Close Collaboration with Biogen on Alzheimer’s Disease Drug*, Public  Citizen (2021), https://www.citizen.org/article/outrage-of-the-month-fdas-inappropriate-close-collaboration-with-biogen-on-alzheimers-disease-drug/.

59 ICER Publishes Final Evidence Report and Policy Recommendations on Aducanumab for Alzheimer’s Disease, Inst. Clin. and  Econ. Rev. (2021), https://icer.org/news-insights/press-releases/icer-publishes-final-evidence-report-and-policy-recommendations-on-aducanumab-for-alzheimers-disease/ (last visited Jan 6, 2022), FAQs | What is ICER?, Inst. Clin. and  Econ. Rev., https://icer.org/who-we-are/faqs/ (last visited Jun 3, 2024). *See also* Angus Liu, *Biogen Halves Aduhelm’s Price and Plots Cost Cuts as Medicare Decision on Controversial Alzheimer’s Drug Nears*, Fierce  Pharma. (2021), https://www.fiercepharma.com/pharma/biogen-halves-aduhelm-price-layoffs-ahead-medicare-decision-controversial-alzheimer-s-drug (last visited Jan. 6, 2022). Some payers indicated they would not cover aducanumab, whereas others announced that they would cover the drug under certain conditions. *See, e.g.*, Joseph Walker, *VA Health System Won’t Cover Biogen’s Alzheimer’s Drug*, The  Wall  Street  Journal, Aug. 12, 2021, https://www.wsj.com/articles/biogens-alzheimers-drug-wont-be-covered-by-va-health-system-11628803740 (last visited Dec. 8, 2021) (Department of Veterans Affairs refused to cover the drug), Denise Myshko, *Some Blues Plans Won’t Cover New Alzheimer’s Therapy Aduhelm*, Formulary  Watch (2021), https://www.formularywatch.com/view/some-blues-plans-won-t-cover-new-alzheimer-s-therapy-aduhelm (Blue Cross Blue Shield plans in North Carolina, Pennsylvania, Michigan, Western New York, and Kansas would not cover aducanumab, but some other Blues plans indicated they would cover the drug with prior authorization), John Tozzi, Angelica Peebles & Anushree Dave, *Insurers Balk at Paying for Biogen’s $56,000-a-Year Alzheimer’s Treatment*, Bloomberg.com, Nov. 18, 2021, https://www.bloomberg.com/news/articles/2021-11-18/insurers-balk-at-paying-for-biogen-alzheimer-s-treatment (surveying twenty-five large insurers and highlighting that Cigna would not cover aducanumab; Humana, a Medicare-focused insurer, would cover the drug for members who resemble those it helped in clinical trials; and Kaiser Permanente would pay for the drug when a member’s physician deemed it to be medically necessary).

60 David Whitrap, *12 Facts to Ponder about the Aduhelm Approval*, Inst. Clin. and  Econ. Rev. (ICER), https://icer.org/news-insights/commentaries/12-facts-to-ponder-about-the-aduhelm-approval/ (last visited Jun 3, 2024).

61 2022 Medicare Parts A & B Premiums and Deductibles/2022 Medicare Part D Income-Related Monthly Adjustment Amounts, cms.gov, Nov. 12, 2021, https://www.cms.gov/newsroom/fact-sheets/2022-medicare-parts-b-premiums-and-deductibles2022-medicare-part-d-income-related-monthly-adjustment.

62 Biogen Announces Reduced Price for ADUHELM® to Improve Access for Patients with Early Alzheimer’s Disease | Biogen, (2021), https://investors.biogen.com/news-releases/news-release-details/biogen-announces-reduced-price-aduhelmr-improve-access-patients (last visited Mar 18, 2024).

63 CMS Opens National Coverage Determination Analysis on Treatment for Alzheimer’s Disease, *supra* note 14.

64
*Id.*

65
*Id.*

66 Medicare Program; Revised Process for Making National Coverage Determinations, 78 Fed. Reg. 48164, *supra* note 9.

67 Medicare Coverage Determination Process, cms.gov, https://www.cms.gov/medicare/coverage/determination-process (last visited Apr. 8, 2023); 42 U.S.C. § 1395y(a) (“In making a national coverage determination … the Secretary shall ensure … that the public is afforded notice and opportunity to comment prior to implementation by the Secretary of the determination… .), 42 U.S.C. § 1395y(l)(3)(B) (‘Beginning on the date the Secretary makes a draft of the proposed [national coverage determination] available … the Secretary shall provide a 30-day period for public comment on such draft’).

68
*Id.*

69 Monoclonal Antibodies Directed Against Amyloid for the Treatment of Alzheimer’s Disease (CAG-00460 N) - Proposed Decision Memo, *supra* note 16.

70 42 U.S.C. § 1395y(a)(1); Medicare Coverage Document - Guidance for the Public, Industry, and CMS Staff: Coverage with Evidence Development, *supra* note 17.

71 42 U.S.C. § 1320b-12(b)(3).

72 Monoclonal Antibodies Directed Against Amyloid for the Treatment of Alzheimer’s Disease (CAG-00460 N) - Proposed Decision Memo, *supra* note 16.

73
*Id.*

74 Alzheimer’s Disease & Down Syndrome, National  Down  Syndrome  Society, https://ndss.org/resources/alzheimers (last visited Apr. 9, 2023).

75 Eric Rubenstein et al., *Medicaid Enrollment and Service Use Among Adults With Down Syndrome*, 4 J ama  Health  Forum. e232320 (2023).

76
*Id.*

77 Monoclonal Antibodies Directed Against Amyloid for the Treatment of Alzheimer’s Disease (CAG-00460 N) - Proposed Decision Memo, *supra* note 16.

78 Monoclonal Antibodies Directed Against Amyloid for the Treatment of Alzheimer’s Disease (CAG-00460 N) - Decision Memo, *supra* note 23 (noting that the remaining 68 comments were withheld due to excessive personal health information content).

79
*Id.*

80 Steven J. Balla et al., *Lost in the Flood?: Agency Responsiveness to Mass Comment Campaigns in Administrative Rulemaking*, 16 Regulation & Governance. 293, 294 (2022) (‘[Mass comment campaigns] generally state directional preferences for or against proposed rules and contain little in the way of legal, economic, scientific, and technical information … .’).

81 CMS-approved studies must also meet several criteria for diverse enrollment, site capacity, study questions, and standards for scientific integrity.

82 Medicare Coverage Policy for Monoclonal Antibodies Directed Against Amyloid for the Treatment of Alzheimer’s Disease, cms.gov, Apr. 7, 2022, https://www.cms.gov/newsroom/fact-sheets/medicare-coverage-policy-monoclonal-antibodies-directed-against-amyloid-treatment-alzheimers-disease.

83
*Id.*

84 Link to the comment portal: https://www.cms.gov/medicare-coverage-database/view/ncacal-public-comments.aspx?NCAId=305.

85
Barney G. Glaser & Anselm L. Strauss, The  Discovery of  Grounded  Theory: Strategies for  Qualitative  Research (4th ed. 2009).

86 Joseph A. Maxwell, *Using Numbers in Qualitative Research*, 16 Qualitative  Inquiry. 475, 479 (2010).

87
*Id.* at 480.

88 Kesselheim included this line in a resignation letter sent to acting FDA Commissioner Janet Woodcock. Kesselheim and Avorn, *supra* note 7.

89
*See*  https://www.medpac.gov. MEDPAC sent a letter to CMS, signed by its Chair Michael Chernew, that stated, in part, “Although the Commission does not normally comment on coverage decisions, we have chosen to do so in this case because of its significance, including the potential fiscal implications, and because the decision invokes coverage with evidence development (CED), which we have discussed in the context of our prescription drug work… [T]he Commission supports CMS’s proposal to implement an NCD that applies CED to cover available anti-amyloid monoclonal antibody drugs, including Aduhelm.” Michael E. Chernew, Paul B. Ginsburg, & James E. Mathews, Ph.D., *Letter from MEDPAC to Chiquita Brooks = LaSure, Administrator, Centers for Medicare & Medicaid Services* (Feb. 10, 2022), https://www.medpac.gov/wpcontent/uploads/2022/02/Feb22_NCD_Monoclonal_Alzheimers_MedPAC_comment_v2_SEC.pdf.

90 For our purposes, a comment was flagged as a ‘duplicate’ when more than five comments shared substantially similar or identical words or phrases.

91 In its own analyses, CMS identified over 5000 comments ‘from form letters, where at least part of the comment contained nearly identical language regarding the evidence and the request for CMS to exclude Aduhelm from coverage.’ Monoclonal Antibodies Directed Against Amyloid for the Treatment of Alzheimer’s Disease (CAG-00460 N) - Decision Memo, *supra* note 23. Elsewhere, CMS reports a large number of letters ‘from stakeholders requesting that patients with Down syndrome and other intellectual and developmental disabilities (IDD) have the same access other patients would have to antiamyloid mAbs.’ *Id.*

92 Public Citizen: About Us, Public  Citizen, https://www.citizen.org/about/.

93 Statement: FDA’s Decision to Approve Aducanumab for Alzheimer’s Disease Shows Reckless Disregard for Science, Severely Damages Agency’s Credibility, Public  Citizen (2021), https://www.citizen.org/news/statement-fdas-decision-to-approve-aducanumab-for-alzheimers-disease-shows-reckless-disregard-for-science-severely-damages-agencys-credibility/.

94
*Id.*

95 Michael A. Carome, *Comments Regarding the Centers for Medicare and Medicaid Services’ National Coverage Determination Analysis for Monoclonal Antibodies Directed Against Amyloid for the Treatment of Alzheimer’s Disease (CAG-00460 N)*, Public  Citizen. (2021), https://www.citizen.org/wp-content/uploads/2598.pdf.

96
*Id.*

97 Social Security Works: About Us, Social  Security  Works, https://socialsecurityworks.org/about/.

98 Submit comments to help protect Medicare from Big Pharma greed!, Social  Security  Works, https://socialsecurityworks.org/2022/02/08/submit-comments-to-help-protect-medicare-from-big-pharma-greed/.

99
*Id.*

100 CMS Comment, National  Down  Syndrome  Society, https://ndss.org/cms-comment (italics in original).

101 At Her Own Pace: Welcome!, At  Her  Own  Pace, https://atherownpace.com/about/.

102 Action Needed by February 9, At  Her  Own  Pace (Feb. 3, 2022), https://atherownpace.com/2022/02/03/action-needed-by-february-9/.

103
*Id.*

104
*Id.*

105
*See* Monoclonal Antibodies Directed Against Amyloid for the Treatment of Alzheimer’s Disease (CAG-00460 N) - Decision Memo, *supra* note 23.

106 42 USC § 1395y(l)(3)(C).

107
*But see* Jenna Wahl & Emily A. Gadbois, *Analysis of the First Round of Public Comments for the National Coverage Determination for Monoclonal Antibodies in the Treatment of Alzheimer’s Disease*, Journal of  Aging & Social  Policy. 1 (2023). Wahl and Gadbois analyzed the 132 comments submitted during CMS’s first public comment period for key trends, themes, and policy implications. *Id.* Comments were solicited through a tracking sheet that posed a series of specific questions that were intended to inform CMS’s analysis while creating its NCD. NCA - Monoclonal Antibodies Directed Against Amyloid for the Treatment of Alzheimer’s Disease (CAG-00460 N) - Tracking Sheet, https://www.cms.gov/medicare-coverage-database/view/ncacal-tracking-sheet.aspx?NCAId=305 (last visited Mar. 18, 2024). Like us, Wahl and Gadbois categorized commentors by stakeholder type and identified key themes across those stakeholders. Wahl & Gadbois. At a high level, our findings are similar, as we found that commentors who supported coverage of mAbs for treatment of AD generally believed aducanumab to be safe and/or effective, and commentors who objected to coverage often highlighted the lack of clinical benefits and associated risks, burdens, and cost inefficiencies. *Id.* One of the biggest differences between their review and our analysis of roughly 100x as many comments is the breakdown of stakeholders who commented; the most frequently represented groups were advocacy organizations, trade associations, and lobbyists; health systems and medical practices; and universities, medical schools, and schools of public health. *Id.* at 5–6. Wahl and Gadbois did not identify patients, Medicare beneficiaries, family members and caregivers, and members of the general public as independent stakeholder groups, indicating that they were not represented frequently enough in this initial comment period to be grouped as their own category. *Id.*

108 For instance, CMS stated that ‘over 5000 were form letters.’ Monoclonal Antibodies Directed Against Amyloid for the Treatment of Alzheimer’s Disease (CAG-00460 N) - Decision Memo, *supra* note 23. We identified approximately 300 more duplicate comments than CMS did, likely because we categorized any comments with a phrase or more that could be identified as stemming from a ‘parent’ comment as a duplicate. We also identified fewer comments in support of full coverage of mAbs than CMS did, a difference of around 200 comments. We defined support of full coverage very narrowly, so only comments that explicitly stated their support for this outcome would have been captured; it is possible that CMS defined this position more expansively. Furthermore, whereas CMS grouped together comments that supported the proposed CED decision and those that opposed any coverage of mAbs for AD, we coded these viewpoints separately because they are not necessarily aligned; supporting coverage under some limited conditions is different from opposing coverage under any conditions.

109 Gideon Lewis-Kraus, *When Dying Patients Want Unproven Drugs*, The  New  Yorker, Jun. 19, 2023, https://www.newyorker.com/magazine/2023/06/26/relyvrio-als-fda-approval.

110 Dunn, *supra* note 56.

111 Largent et al., *supra* note 3.

112 Alzheimer’s Association Welcomes FDA Approval of Aducanumab, Alzheimer’s  Association (2021), https://alz.org/news/2021/alzheimers-association-fda-approval-aducanumab (last visited Nov 16, 2024).

113
*See* Ryan H. Nelson et al., *Bioethics and the Moral Authority of Experience*, 23 Am. J. Bioethics. 12 (2023) (personal experience with an issue has the potential to generate conflicts of interest and unrepresentative perspectives).

114
*See* Alzheimer’s Association Report: 2023 Alzheimer’s Disease Facts and Figures, *supra* note 2.

115 Shana Stites & Jason Karlawish, *Stigma of Alzheimer’s Disease Dementia*, Practical  Neurology. 39 (2018), Emily A. Largent et al., *‘That Would Be Dreadful’: The Ethical, Legal, and Social Challenges of Sharing Your Alzheimer’s Disease Biomarker and Genetic Testing Results with Others*, 8 J. Law  Biosci. 1 (2021).

116
*See* J. H. Karlawish et al., *Caregivers’ Preferences for the Treatment of Patients with Alzheimer’s Disease*, 55 Neurology. 1008 (2000); Jason H. T. Karlawish et al., *Why Would Caregivers Not Want to Treat Their Relative’s Alzheimer’s Disease?*, 51 J. Am. Geriatr  Soc. 1391 (2003). (caregivers are generally willing to slow the progression of their relatives’ dementia even into the severe stage of disease, especially if it can be done without risk to the patient, and their assessment of patient quality of life affects their willingness to use AD-slowing treatments).

117 Alzheimer’s Association Report: 2023 Alzheimer’s Disease Facts and Figures, *supra* note 2.

118 Stites and Karlawish, *supra* note 115.

119 This question has also come up in the IRA implementation process. For recommendations on ensuring patient experiences and preferences are factored into CMS’s decision making, *see, e.g.*, Jennifer Bright et al., *Patient Engagement & Experience Data: Missing Ingredients For CMS’ Successful IRA Implementation*, Health  Affairs  Forefront. (2023), https://www.healthaffairs.org/do/10.1377/forefront.20230515.743661/full/ (last visited Mar. 2, 2024). (‘[A]llowing data submission as the only pathway for public input [in response to initial guidance regarding implementation of the Medicare Drug Price Negotiation Program] is not sufficient to meaningfully receive and incorporate perspectives from the patient community.’).

120 Monoclonal Antibodies Directed Against Amyloid for the Treatment of Alzheimer’s Disease (CAG-00460 N) - Proposed Decision Memo, *supra* note 16.

121 Balla et al., *supra* note 80.

122 Nelson et al., *supra* note 113.

123 Holly Fernandez Lynch & Emily A. Largent, *Considering Tomorrow’s Patients in Today’s Drug Approvals*, 381 Bmj e075000 (2023); Nelson et al., *supra* note 113.

124 Balla et al., *supra* note 80.

125
*Id.* at 306.

126 FAQ and Facts about Down Syndrome, Global  Down  Syndrome  Foundation, https://www.globaldownsyndrome.org/about-down-syndrome/facts-about-down-syndrome/ (last visited Mar. 18, 2024).

127 Monoclonal Antibodies Directed Against Amyloid for the Treatment of Alzheimer’s Disease (CAG-00460 N) - Proposed Decision Memo, *supra* note 16.

128 Research suggests that it is common for noteworthy changes “to occur in contexts in which there are high levels of agreement among participants. Balla et al., *supra* note 80, at 295.

129 Alzheimer’s Association Welcomes FDA Approval of Aducanumab, Alzheimer’s  Association (June 7, 2021), https://alz.org/news/2021/alzheimers-association-fda-approval-aducanumab.

130 Alzheimer’s Association Annual Report 2023, *supra* note 2, at 5.

131
*Id.* at 2.

132 Alicia Ault, *Alzheimer’s Association Denies Conflict in Aducanumab Support*, Medscape (March 8, 2021), https://www.medscape.com/viewarticle/947047.

133
*Id.*

134 Gingery, *supra* note 56.

135 Jon Hu, *Alzheimer’s Drug Approvals Show We Need a Reevaluation of Patient Advocacy*, Stat (Dec. 18, 2023), https://www.statnews.com/2023/12/18/patient-advocacy-alzheimers-lecanemab-aducanamab-fda/ (last visited Nov 16, 2024).

136 David S. Hilzenrath, *Drug Money: In FDA Meetings, ‘Voice’ of the Patient Often Funded By Drug Companies*, Project on  Government  Oversight (2016), https://www.pogo.org/investigations/in-fda-meetings-voice-of-patient-often-funded-by-drug-companies (last visited Nov 16, 2024).

137 Matthew S. McCoy, *Industry Support of Patient Advocacy Organizations: The Case for an Extension of the Sunshine Act Provisions of the Affordable Care Act*, 108 Am J  Public  Health. 1026 (2018). *See also* Susannah L. Rose, *Patient Advocacy Organizations: Institutional Conflicts of Interest, Trust, and Trustworthiness*, 41 The  Journal of  Law, Medicine & Ethics 680 (2013) (‘Good intentions do not immunize [patient advocacy organizations] from bias that may harm their constituents.’).

138 Hu, *supra* note 136.

139 Alzheimer’s Association Welcomes CMS Announcement of National Coverage Determination Analysis on Treatment for Alzheimer’s Disease, Alzheimer’s  Disease and  Dementia. (2021), https://alz.org/news/2021/alzheimers-association-welcomes-cms-announcement-o (last visited Mar. 2, 2024).

140 Harry Johns, *Alzheimer’s Association Statement on CMS Draft Decision*, Alzheimer’s  Association. (2022), https://alz.org/news/2022/alzheimers-association-statement-on-cms-draft-deci (last visited Mar. 2, 2024).

141 Alzheimer’s Association, *Final and Formal Request for Reconsideration of National Coverage Determination (NCD) for Monoclonal Antibodies Directed Against Amyloid for the Treatment of Alzheimer’s Disease (CAG-00460 N)*, (2022), https://alz.org/media/Documents/final-NCD-reconsideration-request.pdf, CMS Treatment Coverage Policy: What It Means for People Living with Alzheimer’s, Alzheimer’s  Association, https://www.alz.org/alzheimers-dementia/treatments/cms-medicare-coverage (last visited Apr. 9, 2023).

142 Johns, *supra* note 141.

143 Laurie McGinley, *Alzheimer’s Groups Vow to Fight Proposed Medicare Restrictions on Controversial Drug*, Wash. Post (Jan. 13, 2022), https://www.washingtonpost.com/health/2022/01/12/alzheimers-drug-aduhelm-will-medicare-cover/.

144 We used the Way Back Machine (web.archive.org) to view the Alzheimer’s Association website as it would have appeared in January 2022.

145 Monoclonal Antibodies Directed Against Amyloid for the Treatment of Alzheimer’s Disease (CAG-00460 N) - Decision Memo, *supra* note 23.

146 There has been growing concern over recent years that sponsors of drug applications granted accelerated approval do not complete their confirmatory trials on schedule; although confirmatory trials are statutorily required, there are few consequences for delays. A recent HHS Office of Inspector General evaluation found that more than one-third of accelerated approval drug applications with incomplete confirmatory trials are past their trials’ original completion date, some by five or more years. U.S. Dept. Health & Human  Servs., Office of  Inspector  General, *Delays in Confirmatory Trials for Drug Applications Granted FDA’s Accelerated Approval Raise Concerns* (2022), https://oig.hhs.gov/oei/reports/OEI-01-21-00401.asp (last visited Mar. 2, 2024); *see also* Anjali D. Deshmukh, Aaron S. Kesselheim & Benjamin N. Rome, *Timing of Confirmatory Trials for Drugs Granted Accelerated Approval Based on Surrogate Measures From 2012 to 2021*, 4 JAMA Health  Forum e230217 (2023) (more than half of confirmatory studies not completed in agreed-upon time). Recent legislative reforms, as well as action at FDA, have aimed at addressing these problems, as discussed below.

147
*See, e.g.*, Kenji Omae et al., *US Food and Drug Administration Accelerated Approval Program for Nononcology Drug Indications Between 1992 and 2018*, 5 Jama  Network  Open e2230973 (2022) (of a cohort study of 48 drugs for non-oncology indications, nine post-approval confirmatory trials failed to verify clinical efficacy but only one indication was withdrawn due to a failed confirmatory trial); *cf.* Ravi B. Parikh et al., *Exposure to US Cancer Drugs With Lack of Confirmed Benefit After US Food and Drug Administration Accelerated Approval*, 9 Jama  Oncology 567 (2023) (among five oncology indications, 26.1% of eligible treatments involved an accelerated approval indication that was subsequently withdrawn due to lack of benefit).

148 U.S. Dept. Health & Human  Servs., Office of  Inspector  General, *supra* note 119.

149 Pam Belluck, *Congressional Inquiry into Alzheimer’s Drug Faults Its Maker and F.D.A.*, N.Y. Times, Dec. 29, 2022, https://www.nytimes.com/2022/12/29/health/alzheimers-drug-aduhelm-biogen.html (last visited Mar. 18, 2024).

150
*See, e.g.*, Peter Johnson, *After Aduhelm, Congress May Revamp Accelerated Approvals*, MMITNetwork (Apr. 7, 2022), https://www.mmitnetwork.com/aishealth/spotlight-on-market-access/after-aduhelm-congress-may-revamp-accelerated-approvals-2/ (‘The FDA’s accelerated approval of Alzheimer’s drug Aduhelm (aducanumab) last year was extremely controversial, prompting harsh criticism and calls for reform of the accelerated approval process itself.’), Jeff Craven, *Expert Proposes Changes to Accelerated Approval Reforms in User Fee Bills*, (2022), https://www.raps.org/News-and-Articles/News-Articles/2022/6/Expert-proposes-changes-to-accelerated-approval-re (last visited Mar. 3, 2024). (discussing the possibility of including changes to accelerated approval as part of the user fee authorization bill).

151 21 USC § 356(c)(3).

152
*Id.*

153
*E.g.*, Robert Califf, *Remarks by Dr Robert Califf on the Reagan Udall Foundation’s Report and Recommendations on Evidence Generation - 11/14/2023*, FDA (2023), https://www.fda.gov/news-events/speeches-fda-officials/remarks-dr-robert-califf-reagan-udall-foundations-report-and-recommendations-evidence-generation (last visited Mar 3, 2024). (‘While [the] post-market phase of medical products is not FDA’s primary responsibility, we do have clear responsibility for safety and evidence to support new indications for medical products,’).

154 Phillips, *supra* note 20.

155 Joint Statement from CMS Administrator Chiquita Brooks-LaSure and FDA Commissioner Robert M. Califf, M.D., on Ensuring Access to Safe and Effective Treatments | CMS, https://www.cms.gov/newsroom/press-releases/joint-statement-cms-administrator-chiquita-brooks-lasure-and-fda-commissioner-robert-m-califf-md (last visited Mar. 18, 2024).

156 FDA’s Califf Defends CMS Drug Coverage Role, Blames Rx Industry Prices For US Health Woes, Pink  Sheet (2023), http://pink.citeline.com/PS148345/FDAs-Califf-Defends-CMS-Drug-Coverage-Role-Blames-Rx-Industry-Prices-For-US-Health-Woes (last visited Mar. 18, 2024).

157 Daval and Kesselheim, *supra* note 21.

158
*Loper Bright Enterprises v. Raimondo*, 603 U.S. ___ (2024).

159
*Id.* For an overview of *Loper Bright*’s possible implications for health regulations, *see* Anna K. Abram et al., *Chevron Runs out of Gas: The Road Ahead for Health Regulations Gets Cloudy After Loper Bright*, Akin  Gump  Strauss  Hauer & Feld LLP (July 8, 2024), https://www.akingump.com/en/insights/alerts/chevron-runs-out-of-gas-the-road-ahead-for-health-regulations-gets-cloudy-after-loper-bright.

160 Wendy E. Parmet, *Loper Bright and the Death of Deference in the Administration of Health Policy*, Health  Affairs  Forefront (July 18, 2024), https://www.healthaffairs.org/do/10.1377/forefront.20240717.933482/full/.

161
*See* Wendy E Wagner, *Administrative Law, Filter Failure, and Information Capture*, 59 Duke L. J. 1321 (2010) (discussing how interested parties can capture the regulatory process by overwhelming it with uncontrolled and excessive information).

162 Daval and Kesselheim, *supra* note 21.

163 Robert King, *New Bill Aims to Reform CMS’ Drug Coverage Authority*, (Dec. 1, 2022), https://www.fiercehealthcare.com/payers/new-bill-aims-reform-cms-drug-coverage-authority-after-controversial-aduhelm-decision.

164
Guide to  Understanding  Health  Technology  Assessment (HTA), (2018), https://icer.org/wp-content/uploads/2020/10/ICER-Guide-to-Understanding-Health-Technology-Assessment-6.19.18.pdf.

165 Monoclonal Antibodies Directed Against Amyloid for the Treatment of Alzheimer’s Disease (CAG-00460 N) - Decision Memo, *supra* note 23.

166
*Id.*

167 Joe Grogan, *Medicare’s ‘Coverage With Evidence Development’: A Barrier to Patient Access and Innovation*, Health  Affairs  Forefront (May 1, 2023), https://www.healthaffairs.org/do/10.1377/forefront.20230428.275268/full/.

168 MLN Connects: 2024-07-11-MLNC, cms.gov (July 11, 2024), https://www.cms.gov/training-education/medicare-learning-network/newsletter/2024-07-11-mlnc.

169
*See, e.g.*, Zosia Kmietowicz & Elisabeth Mahase, *Lecanemab: Benefits of Alzheimer’s Drug Are ‘Just Too Small’ to Justify Cost, Says NICE*, 386 BMJ q1853 (2024).

170 Nirosha Mahendraratnam Lederer & Stacie B. Dusetzina, *Accelerated Approval—An Unexpected Pathway To Value-Based Pricing?* (Aug. 18, 2021), https://www.healthaffairs.org/do/10.1377/forefront.20210812.337699/full/.

171 Ian T. T. Liu, Aaron S. Kesselheim & Edward R. Scheffer Cliff, *Clinical Benefit and Regulatory Outcomes of Cancer Drugs Receiving Accelerated Approval*, 331 Jama 1471, 1471 (2024).

172
*Id.*

173
*See, e.g.*, NORD Position on IRA/CMS Drug Negotiation Price Program, (2023), https://rarediseases.org/driving-policy/public-policy-positions/inflation-reduction-act-ira-nord-point-of-view/ (last visited Mar. 18, 2024); Larry Levitt, *Opinion | The 4 Arguments You Will Hear Against Drug Price Negotiation*, N.Y. Times, Sept. 6, 2023, https://www.nytimes.com/2023/09/06/opinion/medicare-drug-price-negotiation.html.

174
*See, e.g.*, Consuelo H. Wilkins, Suzanne E. Schindler & John C. Morris, *Addressing Health Disparities – Why Minority Recruitment Isn’t Enough*, 77 Jama  Neurology. 1063 (2020).

175 Monoclonal Antibodies Directed Against Amyloid for the Treatment of Alzheimer’s Disease (CAG-00460 N) - Proposed Decision Memo, *supra* note 16.

176 Monoclonal Antibodies Directed Against Amyloid for the Treatment of Alzheimer’s Disease (CAG-00460 N) - Decision Memo, *supra* note 23.

177 Nicholas Florko, *With Its Aduhelm Proposal, Medicare Is Stepping into a Heated Debate about How Best to Improve Equity in Alzheimer’s Treatment*, Stat (Jan. 13, 2022), https://www.statnews.com/2022/01/13/medicare-aduhelm-proposal-debate-equity-alzheimers-treatment/.

178
*Id.*

179
*Id.*

180
*Id.*

181
*Cf.* Adam C. Urato, *FDA: Ignore a Pharma Company’s Deceptive Racial Equity Argument for Keeping Makena, an Ineffective Preterm Labor Drug*, Stat (Sep. 26, 2022), https://www.statnews.com/2022/09/26/fda-ignore-deceptive-racial-equity-argument-for-keeping-makena-on-the-market/ (noting that advocacy organizations—many of which had received industry funding—argued that Makena, a drug with no evidence of benefit to Black women, should be kept on the market to improve racial equity).

182 Monoclonal Antibodies Directed Against Amyloid for the Treatment of Alzheimer’s Disease (CAG-00460 N) - Decision Memo, *supra* note 23.

183
*Id.*

184 For an overview of the benefits and challenges of a timely diagnosis of AD that demonstrates the scarcity of research evidence on early diagnosis, *see* Bruno Dubois et al., *Timely Diagnosis for Alzheimer’s Disease: A Literature Review on Benefits and Challenges*, 49 J. Alzheimers  Dis. 617 (2016).

185 Barak Gaster & Emily A. Largent, *What Makes a Better Life for People Facing Dementia? Toward Dementia-Friendly Health and Social Policy, Medical Care, and Community Support in the United States*, 54 Hastings  Center  Report. S40 (2024).

186 Karlawish, *supra* note 6 (highlighting that the price was seen as too high from the start).

187 Biogen Announces Reduced Price for ADUHELM® to Improve Access for Patients with Early Alzheimer’s Disease | Biogen (2021), https://investors.biogen.com/news-releases/news-release-details/biogen-announces-reduced-price-aduhelmr-improve-access-patients (last visited Mar. 18, 2024).

188
*See* Rachel E. Sachs, Julie M. Donohue & Stacie B. Dusetzina, *Reforming Reimbursement for the US Food and Drug Administration’s Accelerated Approval Program to Support State Medicaid Programs*, 3 Jama  Health  Forum. e224115 (2022) (examining proposed Medicaid reimbursement reform strategies).

189 HHS Secretary Responds to the President’s Executive Order on Drug Prices | CMS, (2023), https://www.cms.gov/newsroom/press-releases/hhs-secretary-responds-presidents-executive-order-drug-prices (last visited Mar. 18, 2024).

190 In October 2023, CMS published an update on this plan, noting that FDA’s new approaches and FDORA authorities may improve timely completion of confirmatory studies, while adding that ‘there is an opportunity for a model that would test ways to encourage completion of confirmatory trials. Additional review and consideration of both the FDA’s new authorities and the shifting mix of products entering the [accelerated approval program] is needed.’ CMS Innovation Center’s One-Year Update on the Executive Order to Lower Prescription Drug Costs for Americans | CMS, (2023), https://www.cms.gov/blog/cms-innovation-centers-one-year-update-executive-order-lower-prescription-drug-costs-americans (last visited Mar. 18, 2024).

191 42 USC 1320f et seq.

192 Rachel E. Sachs & Nicholas Bagley, *Medicare Coverage of Aducanumab — Implications for State Budgets*, 385 N. Engl. J. Med. 2019, 2020 (2021).

193 About MACPAC, MACPAC, https://www.macpac.gov/about-macpac/ (last visited Nov 16, 2024).

194 MACPAC, *Chapter 3: Strengthening Evidence under Medicaid Drug Coverage*, *in*  Report to  Congress on  Medicaid and CHIP (2023), https://www.macpac.gov/wp-content/uploads/2023/03/Chapter-3-Strengthening-Evidence-under-Medicaid-Drug-Coverage.pdf.

195 Monoclonal Antibodies Directed Against Amyloid for the Treatment of Alzheimer’s Disease (CAG-00460 N) - Decision Memo, *supra* note 23.

196 Katelyn N. G. Long et al., *The Role of Hope in Subsequent Health and Well-Being for Older Adults: An Outcome-Wide Longitudinal Approach*, 2 Global  Epidemiology 100018 (2020).

197 Largent et al., *supra* note 3.

198 Fernandez Lynch et al., *supra* note 50.

199 Daniel G. Aaron, *The Fall of FDA Review*, 22 Yale J. Health  Policy, Law, and  Ethics 95 (2023).

200 Amy Kapczynski, *Dangerous Times: The FDA’s Role in Information Production, Past and Future*, 102 Minn. L. Rev. 2357 (2018).

201 Largent et al., *supra* note 3.

202 Zachary Brennan, *Accelerated Approval Will Be ‘the Norm’ for Gene Therapies, FDA’s Peter Marks Says*, Endpoints  News (2024), https://endpts.com/accelerated-approval-will-be-the-norm-for-gene-therapies-fdas-peter-marks-says/ (last visited Nov 16, 2024).

203 Robert Langreth, *Biogen’s Aduhelm Alzheimer’s Drug to No Longer Be Sold, Tested in US - Bloomberg*, Bloomberg.com, Jan. 31, 2024, https://www.bloomberg.com/news/articles/2024-01-31/biogen-will-stop-selling-testing-alzheimer-s-drug-aduhelm (last visited Mar. 18, 2024).

